# Chromatin modifier *developmental pluripotency associated factor 4* (*DPPA4*) is a candidate gene for alcohol-induced developmental disorders

**DOI:** 10.1186/s12916-022-02699-1

**Published:** 2022-12-30

**Authors:** P. Auvinen, J. Vehviläinen, H. Marjonen, V. Modhukur, J. Sokka, E. Wallén, K. Rämö, L. Ahola, A. Salumets, T. Otonkoski, H. Skottman, M. Ollikainen, R. Trokovic, H. Kahila, N. Kaminen-Ahola

**Affiliations:** 1 Environmental Epigenetics Laboratory, Department of Medical and Clinical Genetics, Medicum, University of Helsinki, 00290 Helsinki, Finland; 2 Department of Obstetrics and Gynaecology, Institute of Clinical Medicine, University of Tartu, 50406 Tartu, Estonia; 3 Competence Centre on Health Technologies, 50411 Tartu, Estonia; 4 Research Programs Unit, Stem cells and Metabolism and Biomedicum Stem Cell Centre, Faculty of Medicine, University of Helsinki, 00014 Helsinki, Finland; 5 Division of Obstetrics and Gynaecology, Department of Clinical Science, Intervention and Technology (CLINTEC), Karolinska Institutet, S-171 76 Stockholm, Sweden; 6 Children’s Hospital, Helsinki University Central Hospital, University of Helsinki, 00290 Helsinki, Finland; 7 Faculty of Medicine and Health Technology, Tampere University, 33520 Tampere, Finland; 8 Institute for Molecular Medicine, Finland, FIMM, HiLIFE, University of Helsinki, 00290 Helsinki, Finland; 9 Obstetrics and Gynecology, Helsinki University Hospital, University of Helsinki, 00290 Helsinki, Finland

**Keywords:** Prenatal alcohol exposure, PAE, FASD, DNA methylation, Gene expression, Placenta, Human embryonic stem cells, Germ layers, Endoderm, Mesoderm, Ectoderm, Environmental epigenetics, Embryonic development, *DPPA4*, *DPPA2*, *FOXP2*, *TACR3*

## Abstract

**Background:**

Prenatal alcohol exposure (PAE) affects embryonic development, causing a variable fetal alcohol spectrum disorder (FASD) phenotype with neuronal disorders and birth defects. We hypothesize that early alcohol-induced epigenetic changes disrupt the accurate developmental programming of embryo and consequently cause the complex phenotype of developmental disorders. To explore the etiology of FASD, we collected unique biological samples of 80 severely alcohol-exposed and 100 control newborns at birth.

**Methods:**

We performed genome-wide DNA methylation (DNAm) and gene expression analyses of placentas by using microarrays (EPIC, Illumina) and mRNA sequencing, respectively. To test the manifestation of observed PAE-associated DNAm changes in embryonic tissues as well as potential biomarkers for PAE, we examined if the changes can be detected also in white blood cells or buccal epithelial cells of the same newborns by EpiTYPER. To explore the early effects of alcohol on extraembryonic placental tissue, we selected 27 newborns whose mothers had consumed alcohol up to gestational week 7 at maximum to the separate analyses. Furthermore, to explore the effects of early alcohol exposure on embryonic cells, human embryonic stem cells (hESCs) as well as hESCs during differentiation into endodermal, mesodermal, and ectodermal cells were exposed to alcohol in vitro.

**Results:**

*DPPA4, FOXP2,* and *TACR3* with significantly decreased DNAm were discovered—particularly the regulatory region of *DPPA4* in the early alcohol-exposed placentas. When hESCs were exposed to alcohol in vitro, significantly altered regulation of *DPPA2*, a closely linked heterodimer of *DPPA4*, was observed. While the regulatory region of *DPPA4* was unmethylated in both control and alcohol-exposed hESCs, alcohol-induced decreased DNAm similar to placenta was seen in in vitro differentiated mesodermal and ectodermal cells. Furthermore, common genes with alcohol-associated DNAm changes in placenta and hESCs were linked exclusively to the neurodevelopmental pathways in the enrichment analysis, which emphasizes the value of placental tissue when analyzing the effects of prenatal environment on human development.

**Conclusions:**

Our study shows the effects of early alcohol exposure on human embryonic and extraembryonic cells, introduces candidate genes for alcohol-induced developmental disorders, and reveals potential biomarkers for prenatal alcohol exposure.

**Supplementary Information:**

The online version contains supplementary material available at 10.1186/s12916-022-02699-1.

## Background

Prenatal alcohol exposure (PAE) is associated with a broad spectrum of permanent structural, physiological, neurocognitive, and behavioral disorders of the exposed, often growth-restricted offspring [1]. Fetal alcohol spectrum disorders (FASD) are a consequence of PAE and an umbrella term for all alcohol-induced developmental disorders. PAE is one of the most harmful environmental factors affecting permanently 3–5% of individuals in the Western world [2].

Several lines of evidence suggest that the epigenome of developing embryo is sensitive to environmental effects in the early pregnancy, during the dynamic period of epigenetic reprogramming [3, 4]. Alcohol-induced epigenetic alterations have been observed in the offspring of our early PAE mouse model [5, 6] as well as human and mouse embryonic stem cells [7, 8]. Those early epigenetic changes could affect gene regulation and consequently developmental programming. Depending on the function of the cell or tissue types, they could contribute to the complex phenotype of FASD.

To explore the etiology of FASD, we have collected placental samples from PAE and control newborns at birth. Placenta is an accessible human tissue and a promising implement for identifying the effects of intrauterine environment on embryonic development, including neuronal development [9, 10]. Here, by performing genome-wide DNA methylation (DNAm) analysis of placenta, we discovered a candidate gene *developmental pluripotency associated factor 4* (*DPPA4*), which was hypomethylated particularly in the early alcohol-exposed placentas. *DPPA4* functions as a heterodimer with *developmental pluripotency associated factor 2* (*DPPA2*) and both proteins are required for efficient binding and chromatin remodeling [11]. By modifying chromatin structure, these epigenetic priming factors facilitate transition between pluripotency and differentiation [11–13], which makes them plausible candidate genes for developmental disorders. Both genes are located in tandem on chromosome 3q13.13, they are regulated by promoter DNAm in mouse, and are expressed for a short time in the beginning of embryonic development [14]. To explore the effects of early alcohol exposure on human embryonic cells, and more specifically on the regulation of *DPPA2* and *DPPA4*, we performed genome-wide DNAm and gene expression analyses for in vitro alcohol-exposed human embryonic stem cells (hESCs). Furthermore, hESCs were in vitro alcohol-exposed during differentiation into the endodermal, mesodermal, and ectodermal cells.

Alcohol-induced epigenetic changes in the first embryonic cells could be fixed in persistent cellular memory and mitotically transmitted to different cell and tissue types. Indeed, variety of PAE-associated DNAm changes in peripheral blood [15] and buccal epithelial cells (BECs) [16, 17] of children with FASD have been observed in previous genome-wide studies. Therefore, we examined whether PAE-associated epigenetic alterations can be detected not only in the extraembryonic placenta, but also in embryonic white blood cells (WBCs) from cord blood or BECs of the same newborns. Those changes could be the first fingerprints of PAE, potential future biomarkers for FASD, which would enable early diagnosis and personalized support for the development of the affected children.

## Methods

### Epigenetics of FASD (epiFASD) cohort

Women (*n* = 80) with substantial alcohol consumption were recruited to this study in a special outpatient clinic for pregnant women with substance use problems in Helsinki University Hospital, Finland during the years 2013–2020 (Table 1 and Additional file 1: Table S1). The timing of maternal alcohol consumption was registered using self-reported information. To avoid specific individual level data, the timing of consumption is presented in three categories according to pregnancy trimesters (Additional file 1: Table S1 and Additional file 2: Fig. S1). The amount of maternal alcohol consumption was registered using self-reported information: Alcohol Use Disorders Identification Test (AUDIT) or the number of alcohol units consumed per week (ad) (one unit is 12 g of ethyl alcohol). A 10-item screening tool AUDIT, developed by the World Health Organization, estimates alcohol consumption, drinking behavior, and alcohol-related problems [18]. Maternal alcohol consumption is presented in three categories according to AUDIT scores or ad [19, 20]. However, self-reported information (not categories) about timing of drinking, AUDIT scores, and ad were used in statistical analyses. The mothers who consumed alcohol up to gestational week (GW) 7 at maximum were selected in the early PAE subgroup. Only samples with the most specified information about the maternal alcohol consumption were included (27 newborns) (Additional file 1: Table S1). According to the chart reviews, the majority, 66 (82.5%) mothers of all PAE newborns smoked, and 18 (22.5%) mothers used antidepressants or antipsychotic medication during the pregnancy. Five (6.3%) mothers used gestational diabetes mellitus medication. Four mothers had thyroid diseases, two had antihypertensive medication, and one had preventive medication for herpes. One mother had FAS diagnosis. One mother was an occasional user of stimulants and one of cannabis. Fifteen (18.8%) of the deliveries were cesarean sections (CS). Due to the preterm premature rupture of membranes, two newborns were preterm. One of the PAE newborns had two thumbs in one hand, and three had cleft lip. One newborn was Asian, one was Caucasian (other than Finnish), and two were of African origin. One’s mother was Caucasian (other than Finnish), and one had African origin father. Other newborns were children of Finnish, Caucasian parents.

**Table 1:** General characteristics of PAE and control newborns as well as their mothers included in the phenotype analysis

**Newborns**	**Sex**	**Gestational age**		**Birth weight**		**Birth length**		**HC**		**Placental weight**
	(male/female)	(weeks +days)		(g)	SD	(cm)	SD	(cm)	SD	(g)
		all	without CS/IL							
**Control (*n*** **= 100)** **mean (±SD)**	52.0/ 48.0%	40 +3 (±1 +0)	40 +4 (±1 +0) *n* = 90	3661 (±366)	0.07 (±0.9)	51 (±2)	−0.24 (±0.7)	35.5 (±1.3)	0.25 (±0.9)	626.5 (±126.4)
**PAE (*n*** **= 80)** **mean (±SD)**	42.5/57.5%	39 +4 (±1 +5)	39 +4 (±1 +6) *n* = 60	3327 (±554)	−0.23 (±0.1)	49 (±3)	−0.50 (±0.8)	34.4 (±1.9)	−0.14 (±1.2)	596.6 (±136.7)
**Difference between groups,** ***P*-value**	0.231^(3)^	<.001^(1)^	<.001^(1)^	<.001^(1)^	0.030^(1)^	<.000^(1)^	0.044^(2)^	<.000^(1)^	0.012^(2)^	0.083^(1)^
**Early PAE (*n*** **= 27)** **mean (±SD)**	44.4/55.6%	40 +1 (±1 +5)	40 +2 (±1 +4) *n* = 23	3526 (±440)	−0.06 (±0.8)	50 (±2)	−0.42 (±0.8)	34.5 (±1.7)	−0.24 (±1.2)	620.7 (±160.1)
**Difference between groups,** ***P*-value**	0.522^(3)^	0.804^(1)^	0.884^(1)^	0.147^(2)^	0.485^(2)^	0.267^(1)^	0.333^(2)^	<.001^(1)^	0.016^(2)^	0.843^(1)^
**Mothers**	**AUDIT score**	**Alcohol consumption**	**Smoking**	**Age**	**Parity**	**Pre-pregnancy BMI**	**Gestational weight gain**			
		(ad)	(smokers/non-smokers	(years)		(kg/m^2^)	(kg)			
**Control (*n*** **= 100)** **mean (±SD)**			0.0/100%	31.6 (±4.6)	0.55 (±0.7)	22.8 (±3.5) *n* = 99	15.1 (±3.8) *n* = 88			
**PAE (*n*** **= 80)** **mean (±SD)**	18.5 (±8.4) *n* = 44	24.8 (18.7) *n* = 26	82.5/17.5%	29.5 (±6.8)	0.66 (±1.3)	25.4 (±5.9) *n* = 67	12.4 (±6.7) *n* = 56			
**Difference between groups,** ***P*-value**				0.017^(1)^	0.269^(1)^	0.008^(1)^	0.008^(2)^			
**Early PAE (*n*** **= 27)** **mean (±SD)**	19.9 (±7.2) *n* = 18	31.5 (15.8) *n* = 8	74.1/25.9%	27.1 (±5.9)	0.26 (±0.7)	24.3 (±4.9) *n* = 22	15.7 (±6.9) *n* = 16			
**Difference between groups,** ***P*-value**				0.001^(2)^	0.017^(1)^	0.212^(1)^	0.711^(2)^			

Differences in weight, length, and head circumference (HC) of the newborns were calculated using both anthropometric measures and the standard deviations (SDs) of measures based on international growth standards adjusted for gestational age at birth and gender. Data presented as mean ±SD. *P*-values for PAE and control newborns as well as for early PAE and control newborns were calculated using Mann-Whitney *U* test (1) or two-tailed Student’s *t* test (2) and for the proportion of males and females between the groups using Pearson chi-square (3). Differences in gestational age were calculated for all newborns and for newborns delivered spontaneously without cesarean section (CS) and induction of labor (IL). ILs due to prolonged control pregnancies (42+1) were not excluded. AUDIT: alcohol use disorders identification test before pregnancy and ad: alcohol units consumed per week

The control samples (*n* = 100), collected during the years 2013–2015 in Helsinki University Hospital, Finland, were from newborns of healthy Finnish, Caucasian mothers who did not use alcohol or smoke during pregnancy according to their self-reported information (Additional file 1: Table S2). Ten (10%) of the deliveries were CSs.

The information about the samples included in the early PAE subgroup as well as in each analysis is shown in the Additional file 1: Tables S1 and S2, and the general characteristics of the participants in each analysis are shown in the Additional file 1: Table S3.

### Sample collection

Biological samples (placental biopsies, WBCs from umbilical cord blood, and BECs) of newborns were collected immediately after delivery. When this was not possible, placenta was stored in the fridge for a maximum of 12 h and only DNA was extracted for further analyses. The placental biopsies (1 cm^3^) were collected from the fetal side of the placenta within a radius of 2–4 cm from the umbilical cord, rinsed in cold 1× PBS, and stored in RNAlater® (Thermo Fisher Scientific) at −80 °C. WBCs were extracted from umbilical cord blood as soon as possible, at latest 16 h after birth as described previously [21]. BEC samples were collected by rubbing buccal swabs (SK-3S, Isohelix or Catch-All™ Sample Collection Swab, Epicentre Biotechnologies) 20 times firmly against the inside of the newborn’s cheek and stored at −80 °C.

Birth weight (g), birth length (cm), and head circumference (HC) (cm) were examined using international growth standards, the Fenton Preterm Growth Chart by PediTools [22], in which the gestational age at birth and sex are considered when calculating the standard deviation (*z*-score) of birth measures (SD of birth measures) [23].

### hESC and differentiation experiments

#### hESC culture and alcohol treatment

Alcohol concentration of 70 mM, which corresponds to the blood alcohol concentration of a heavy drinker [24], was chosen according to a previous publication [25]. hESC lines H1 (WA01, male) and Regea08/017 (female) were cultured in E8 or in E8 Flex Medium (Gibco) on Matrigel (Corning) coated plates at 37 °C and 5% CO_2_. Culture media was routinely replaced every day (every second or third day in the case of E8 Flex Medium), and cells were passaged using 0.5 mM EDTA. For the alcohol treatment, the medium was supplemented with alcohol (≥ 99.5 p-% ethanol) at a final concentration of 70 mM 48 h before the cells reached confluency and were cross-linked for chromatin immunoprecipitation (ChIP) or collected for DNA and RNA extractions. Due to alcohol evaporation, the culture media with alcohol were replaced after treatment of 24 h.

#### Germ layer cell differentiation and alcohol treatment

H1 cells cultured in E8 Medium on Matrigel plates were differentiated into the endodermal, mesodermal, and ectodermal cells by using the STEMdiff™ Trilineage Differentiation Kit (StemCell Technologies, Inc.). Cells were seeded on a Matrigel-coated 6-well plates at 250,000 cells/well for the mesoderm, 1 million cells/well for the endoderm, and 1.5 million cells/well for the ectoderm, and differentiated according to the manufacturer’s instructions. The cells were supplemented with 10 μM Y-27632 for the first 24 h after seeding, and the mediums were changed daily. For the alcohol-exposed wells, the medium was supplemented with alcohol at a final concentration of 70 mM. After 5 or 7 days, the cells were collected for DNA and RNA extractions. The differentiation was confirmed by 3’mRNA sequencing (mRNA-seq) analysis, and expression profiles of gene characteristic for specific germ layers were analyzed (Additional file 2: Fig. 2).

### DNA and RNA extractions

Placental genomic DNA was extracted from one to four (3.7 on average) pieces of placental tissue samples using commercial QIAamp Fast DNA Tissue Kit (Qiagen) or standard phenol-chloroform protocol. WBC DNA was extracted using QIAamp Fast DNA Tissue Kit or AllPrep DNA/RNA/miRNA Universal Kit (Qiagen) and BEC DNA using BuccalPrep Plus DNA Isolation Kit (Isohelix). Placental RNA was extracted from the same pieces as DNA (2.9 on average) by AllPrep DNA/RNA/miRNA Universal Kit, and the same kit was used for DNA and RNA extraction from hESCs and differentiated hESCs. RNA quality was assessed using an Agilent 2100 Bioanalyzer (Agilent Technologies, Inc.), which was provided by the Biomedicum Functional Genomics Unit (FuGU) at the Helsinki Institute of Life Science and Biocenter Finland at the University of Helsinki.

### DNAm microarrays

Genomic DNA (1000 ng) from available placental (all PAE *n* = 69, early PAE *n* = 27, and control *n* = 66), hESC (H1 and Regea08/017: *n* = 4/condition, respectively), and differentiated H1 hESC (each germ layer: *n* = 4/condition) samples was sodium bisulfite-converted using the Zymo EZ DNAm™ kit (Zymo Research), and genome-wide DNAm was assessed with Infinium Methylation EPIC BeadChip Kit (Illumina) following a standard protocol.

#### Genome-wide DNAm analysis

The raw DNAm dataset was pre-processed, quality controlled, and filtered using ChAMP R package [26] with default settings. The detection *P*-value cutoff was set at *P* = 0.01, and probe bead count > 3 in at least 95% of samples. All probes and samples passed these QC thresholds and were included in the subsequent steps. The data filtering steps included the removal of probes located in sex chromosomes and probes binding to polymorphic and off-target sites [27]. Finally, Type-I and Type-II probes were normalized using the BMIQ method. For placental samples, probes located in Finnish-specific SNPs (SNPs which overlap with any known SNPs with global minor allele frequency (MAF) and MAF in a Finnish population > 1%) were removed as described previously [28]. Population-specific masking and SNP information was obtained from Zhou et al. [29]. Subsequently, a total of 588,781 probes of placental and 800,002 probes of hESC samples were retained for further downstream analysis. Potential batch effects caused by technical factors and biological covariates were studied from *singular value decomposition (SVD) plots*. For placental samples, the correction for batch effect was performed by the Empirical Bayes method using the R package ComBat [30]. Genome-wide DNAm analysis by using M-values was performed by R package Limma [31], and the model for placental samples was adjusted to consider biological covariates sex and smoking. The *λ* values and Quantile-Quantile (Q-Q) plots are shown in Additional file 2: Fig. S3a. Planet R package [32] was used to count placental cell-type fractions by CIBERSORT method and used as an adjusting factor in the model. For hESCs, a mixed linear model was built by using humanzee R package [33] to remove sample-specific random effect and to adjust cell line. *β*-values were used for visualization and interpretation of the results and to construct the DNAm profiles of differentiated cells.

CpGs were considered as significant (hereafter differentially methylated positions, DMPs) when DNAm difference was greater than 5% (Δ*β* ≤ −0.05 and Δ*β* ≥ 0.05) and false discovery rate (FDR)-corrected *P*-value smaller than 0.05. Benjamini-Hochberg procedure was used to control for FDR. Annotation information of the University of California, Santa Cruz (UCSC) database about CpG sites were obtained and merged to corresponding CpG sites from IlluminaHumanMethylationEPICanno.ilm10b4.hg19 R package [34], which is based on the file “MethylationEPIC_v-1-0_B4.csv” from Illumina [35]. If the UCSC database location information was missing, DMP was marked as “unknown.” In the case of multiple location entries, group “others” was used. Otherwise, the following abbreviations were used: TSS1500: 1500 bp upstream of transcription start site, TSS200: 200 bp upstream of TSS, UTR: untranslated region, N_shelf: north shelf, N_shore: north shore, S_shore: south shore, S_shelf: south shelf.

#### Sensitivity analysis for DMPs

To test the sensitivity of DMPs, only non-smoking samples (66 controls and 11 PAE) were selected to the differential DNAm analysis, which was adjusted only for sex. The sensitivity analysis was also performed for the samples of early PAE subgroup (66 controls and six PAE).

#### Sensitivity analysis for candidate genes

Samples (66 control and eight PAE) for the sensitivity analysis of three candidate genes (*DPPA4*, *FOXP2*, and *TACR3*) were selected by excluding the effects of smoking and extraction methods. The differential DNAm analysis was adjusted for sex, maternal age, mode of delivery, and parity as covariates according to the SVD plot (Additional file 2: Fig. S4a). After these adjustments, the group (control/PAE) was the most significant factor in the SVD plot, whereas other factors (AUDIT, HC, birth weight, birth length) associated with PAE and therefore cannot be adjusted for. The sensitivity analysis was also performed for the samples in the early PAE subgroup (66 controls and four PAE) (Additional file 2: Fig. S4b). The *λ* values of the sensitivity analyses were improved compared to DMP analyses, as expected (Additional file 2: Fig. S3b).

#### Differentially methylated region (DMR) analysis

DMRcate R package was used for analyzing DMRs [36]. The method uses minimum description length for detecting region boundaries in DMR identification. DMRcate was adjusted to determine probes (≥ 3) in a region with maximal allowed genomic distance of 1000 bp containing at least one CpG with Δ*β* ≤ −0.05 or Δ*β* ≥ 0.05. Further, FDR < 0.05 was defined to describe the DMR with significance.

#### Enrichment analysis of DMPs at genomic locations

Enrichment of DMPs at different genomic locations relative to gene (TSS1500, TSS200, 5′UTR, 1stExon, Body, 3′UTR, Others, Unknown) and relative to CpG island (N_Shelf, N_Shore, Island, S_Shore, S_Shelf, OpenSea) were calculated for both placenta and hESCs and results were compared to the proportions of probes in the EPIC Array by Fisher’s exact test of homogeneity followed by pairwise comparison post hoc test.

#### Genome-wide average DNAm (GWAM)

After quality filtering steps, remaining 588,781 probes were used to calculate the genome-wide average DNAm levels of placental samples, and 800,002 probes were used for GWAM [37] of hESCs.

#### Global DNAm

Filtered and corrected DNAm data was used to predict DNAm in Alu, LINE1, and LTR using Random Forest-based algorithm implemented by REMP R package [38] as a proxy for global DNAm level. Less reliable predicted results were trimmed according to quality score threshold 1.7 and missing rate 0.2 (20%).

#### Pathway analysis

Enrichment analysis was performed for significant DMPs by *gometh* function in missMethyl R package [39], which considers the different number of probes per gene present on the EPIC array and CpGs that are annotated to multiple genes. missMethyl was set to use the Gene Ontology (GO) knowledgebase as the source for identifying significantly enriched biological process (BP) terms from genes which contained at least one significant DMP. Pathway analysis was also performed for significant DMRs by *goregion* function in missMethyl R package and GO:BP knowledgebase was used as a source. For the enrichment analysis, DMRs with two CpG sites were also included. None of the GO terms were significant after FDR correction and therefore the terms with the nominal *P*-value < 0.05 were reported.

### EpiTYPER

To validate and replicate the findings from the EPIC microarrays, DNAm profiles of target genes (*DPPA4, FOXP2,* and *TACR3*) were measured by MassARRAY EpiTYPER (Agena Bioscience, Inc.) in placental tissue, WBCs, BECs, and hESCs. Samples of 16 PAE and 14 control newborns were chosen for the *DPPA4* target gene and 10 PAE and 9 control newborns for other target genes. In hESCs, two biological replicates of alcohol-exposed and control cells of both H1 and Regea08/017 cell lines were used for the analysis. First, genomic DNA (500–1000 ng) was subjected to sodium bisulfite conversion using EZ-96 DNA Methylation™ kit (Zymo Research). PCR amplification was performed in three independent 10 or 15 μl reactions using HotStar PCR kit (Qiagen) following the provider’s instructions. Primers for the target regions were designed using EpiDesigner software [40] incorporating CpGs chosen for each target according to the microarray analysis. Primers for *TACR3* DMP cg18538958 with the largest effect size were unable to design and therefore a correlating probe cg16461251 (*r* = 0.973, *P* < 0.001, *n* = 136, Spearman’s rank correlation) was selected for the analysis. Primers and PCR protocols for each target sequence are presented in Additional file 1: Tables S4 and S5. Owing to the proximity of two CpGs in one unit in *FOXP2*, they were analyzed together as the mean DNAm value. Technical replicates showing > 5% from the median value were excluded, and the DNAm values from the remaining two replicates were used in the further analyses. Samples with two or three unsuccessful replicates were excluded.

### 3’mRNA sequencing (mRNA-seq) analysis

#### Differential expression analysis

Drop-seq pipeline [41] was used to construct the mRNA-seq count table for available placental (all PAE *n* = 64, early PAE *n* = 23, and control *n* = 41), hESC (H1: alcohol-exposed *n* = 7 and control *n* = 6, Regea08/017: alcohol-exposed and control *n* = 6, respectively), and differentiated male H1 hESC (control/alcohol-exposed endoderm *n* = 4/3, mesoderm *n* = 3/3, and ectoderm *n* = 4/4) RNA samples provided by FuGU. A total of 38,434 transcripts were identified for downstream analysis of placental and 30,081 transcripts of hESC samples. Principal component analysis (PCA) implemented in DESeq2 [42] was used to identify batch effects, and ComBat-seq [43] was used to adjust separate mRNA-seq batches. Differential expression analysis was performed by DESeq2 R package, with model adjusting for smoking and sex for placental samples and with model adjusting cell line for hESCs. Genes were considered as differentially expressed when FDR-corrected *P*-value was < 0.05. Benjamini-Hochberg procedure was used to control for FDR. To validate the hESC differentiation into the germ layer cells, normalized counts of marker genes were used in heatmap visualization (Additional file 2: Fig. S2).

#### Pathway analyses

*enrichgo* function in R package clusterProfiler version 4.0 [44] was used to perform gene-set enrichment analysis for significant differentially expressed genes. The GO knowledgebase was used as the source for identifying significantly enriched BP terms (FDR-corrected *q*-value < 0.05). Benjamini-Hochberg procedure was used to control for FDR.

### Correlation analysis

Normalized genome-wide DNAm data was compared to similarly adjusted mRNA-seq data to discover genes, which DNAm changes correlate with mRNA expression in the placenta and hESCs. A total of 53 PAE and 39 control placental samples as well as eight alcohol-exposed and eight control hESC samples (H1 and Regea08/017: *n* = 4/condition, respectively), of which DNAm and mRNA expression data were available, was used. DNAm and expression data were adjusted to include only the same identified genes between the analyses. For placental data, a total of 126,810 probes were clustered according to 14,635 genes, which were identified from the mRNA-seq data. For hESCs, 106,341 probes were clustered according to 14,051 genes. MethylMix version 2.20.0. R package [45] was used to perform correlation analysis.

### Common genes in genome-wide DNAm and mRNA-seq analyses

The gene name annotation information from DNAm (all differentially methylated CpGs with FDR < 0.05) and mRNA-seq (FDR < 0.05) analyses of placenta and hESCs were used to explore the common genes that associate significantly with alcohol exposure. When CpG was annotated to multiple genes, the first UCSC gene name were chosen. If the UCSC gene name was missing, the GENCODE database information was used. GO:BP enrichment analysis of the common genes was performed by R package clusterProfiler version 4.0 (see mRNA-seq pathway analysis).

### ChIP-qPCR

ChIP was performed for ~5 million hESCs as described in Schmidt et al. [46] with some modifications using H1 and Regea08/017 cell lines. Two replicates of both cell lines were used, which is four replicates of alcohol-exposed and control ChIP samples in total. Briefly, cells were cross-linked using 1% formaldehyde and sonicated with Bioruptor® Pico sonication device (Diagenode) using optimized parameters 4 cycles of 30s on/90s off to generate DNA fragments of 300–600 bp. For immunoprecipitation, 0.75 mg of Dynabeads™ Protein G magnetic beads (Invitrogen) were first incubated with 5 μg of antibodies against H3K4me3, H3K4me2, H3K9ac, and H3 (Additional file 1: Table S6). Subsequently, the shared chromatin was incubated with antibody-bound protein G beads overnight at 4 °C with rotation. The protein-DNA complexes were then washed, eluted, reverse cross-linked, and treated with Proteinase K and RNase A (Thermo Scientific). Finally, the DNA was purified using QIAquick PCR Purification Kit (Qiagen) and used as a template for quantitative PCR (qPCR). The qPCR was performed in triplicates of 10 μl reactions using SsoAdvanced™ Universal SYBR® Green Supermix (Bio-Rad Laboratories) according to the manufacturer’s instructions. The enrichment was normalized against input and further against total histone H3 enrichment. To compare the enrichment between alcohol-exposed and control samples, the data were also normalized against a negative control region designated as “Gene desert.” Target sequences were designed to incorporate regions at *DPPA2* and *DPPA4* enriched with histone modifications of interest in the H1 hESC line according to the Encyclopedia of DNA Elements (ENCODE) [47]. Primers for the target sequences were designed using Primer3 [48], and primers for the negative control region were obtained from a previous publication [49]. Primers and location of amplicons in the genome, as well as qPCR protocol, are provided in Additional file 1: Tables S4 and S5.

### Statistical analysis

All statistical analyses were conducted in R version 4.2.0 [50], IBM SPSS Statistics for Windows, version 27.0 (IBM Corp.), or GraphPad Prism 9 software (GraphPad Software, Inc.). All data are expressed as the mean with ±SD for a normal distribution of variables. Statistical analyses were performed as described in the figure legends or in the relevant sections. Pearson correlation coefficient was used for normally distributed DNAm data; otherwise, Spearman’s rank correlation coefficient was used.

## Results

### Characteristics of epiFASD cohort

General characteristics of epiFASD cohort including 80 PAE and 100 control newborns as well as their mothers were compared (Table 1, Additional file 1: Tables S1 and S2). PAE newborns had significantly smaller birth weights (SD), lengths (SD), and HCs (SD) compared to control newborns (*P* = 0.030, Mann-Whitney U, *P* = 0.044 and *P* = 0.012, respectively, Student’s *t* test). Moreover, to explore more specifically the effects of early PAE on the phenotype as well as placental epigenome and gene expression, we selected 27 newborns whose mothers had consumed alcohol up to GW 7 at maximum to the separate analyses. Notably, also the newborns in this early PAE subgroup had significantly smaller HCs (SD) compared to controls (*P* = 0.016, Student’s *t* test).

When potential correlations between changes in placental weight (g), birth measures (SDs), and maternal alcohol consumption determined by AUDIT scores [18] or ad were calculated between the PAE as well as early PAE newborns and controls, a negative correlations between AUDIT scores and birth length (SD) were found (*r* = -0.505, *P* < 0.001, *n* = 44, *r* = -0.576, *P* = 0.012, *n* = 18, respectively, Spearman’s rank correlation). The gestational age was significantly shorter in PAE pregnancies compared to the controls (*P* < 0.001, Mann-Whitney *U*). Furthermore, the mothers of PAE newborns had significantly higher pre-pregnancy BMI, but gained significantly less weight during pregnancy than the mothers of control newborns (*P* = 0.008, Student’s *t* test and *P* = 0.008, Mann-Whitney *U*, respectively). However, the weight gain in both groups was within the recommended range [51].

### Effects of PAE on genome-wide DNAm in the placenta

**Fig. 1:**
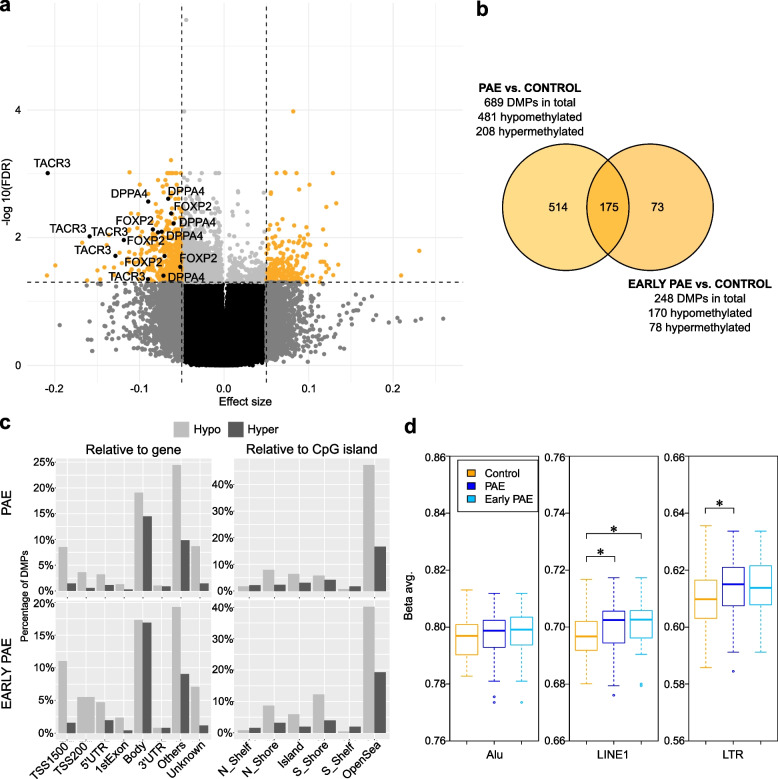
PAE-associated differential DNAm in the placenta. **a** Volcano plot showing the distribution of associations between placental CpG sites and PAE. Horizontal line marks FDR 0.05 and vertical line marks effect size ± 0.05. **b** Venn diagram showing the number of PAE-associated DMPs, which are in common between all PAE placentas and the early PAE subgroup. **c** Genomic location of PAE- and early PAE-associated DMPs in relation to gene and CpG island in the placenta. DMPs were divided to hypo- and hypermethylated subgroups, which were further grouped according to the genomic location based on UCSC database. If the location information was missing, DMP was marked as “unknown.” In the case of multiple location entries, group “others” was used. **d** Effects of PAE on global placental DNAm level predicted by Alu, LINE1, and LTR repetitive regions in all PAE placentas and in the early PAE subgroup. *P < 0.05, two-tailed Student’s *t* test. Control *n* = 66, PAE *n* = 69, and early PAE *n* = 27. Abbreviations TSS1500: 1500 bp upstream of transcription start site, TSS200: 200 bp upstream of TSS, UTR: untranslated region, N_shelf: north shelf, N_shore: north shore, S_shore: south shore, S_shelf: south shelf

We used Illumina’s Infinium MethylationEPIC microarrays to identify PAE-associated genome-wide DNAm alterations in 69 PAE and 66 control full-term placentas (Samples in Additional file 1: Tables S1 and S2, and general characteristics in Additional file 1: Table S3). By adjusting for batch, sex, and smoking covariates, the analysis resulted in 2538 significantly differentially methylated CpG sites (2138 hypomethylated and 400 hypermethylated) with FDR < 0.05 (Fig. 1a,b and Additional file 1: Table S7). To separate the most prominent changes and to minimize false positive hits (*λ* = 1.69, Additional file 2: Fig. S3a), we focused on the CpG sites with DNAm difference of ≥ 5% between PAE and control placentas, which are termed as differentially methylated positions (DMPs). There were 689 DMPs associated with PAE (FDR < 0.05, Δ*β* ≤ −0.05 and Δ*β* ≥ 0.05), of which 481 were hypomethylated and 208 hypermethylated. The PCA performed for DMPs indicated that although observed alterations associate with PAE, smoking appeared to be a strong confounding factor (Additional file 2: Fig. S5).

The analysis revealed five hypomethylated DMPs in *DPPA4.* Furthermore, *transcription factor forkhead box P2* (*FOXP2*), which is needed for the development of speech regions in the brain during embryogenesis [52, 53], had six hypomethylated DMPs, and *Tachykinin Receptor 3* (*TACR3 or neurokinin 3 receptor, NK3R*) expressed in the central nervous system had five hypomethylated DMPs. Interestingly, genetic polymorphisms in *TACR3* have been previously associated with alcohol and cocaine addiction [54], and hypomethylation of the promoter region in blood is linked to repeated cocaine administration in marmoset monkeys [55]. The observed DMPs were mainly located in regulatory regions and in the first exons (Additional file 1: Table S7). Furthermore, all three candidate genes were associated with PAE in sensitivity analyses, in which known potential cofounding factors were excluded (Additional file 1: Tables S8 and S9), and their effect sizes were consistently altered in all performed analyses (Additional file 2: Fig. S6).

In addition to testing for associations for each CpG separately, we tested for differentially methylated regions (DMRs) defined as a region with maximal allowed genomic distance of 1000 bp containing three or more CpGs of which at least one CpG with a Δ*β* ≤ −0.05 or Δ*β* ≥ 0.05. A total of 112 DMRs were observed, including highly significantly hypomethylated DMRs in *DPPA4* (6 CpGs), *FOXP2* (7 CpGs), and *TACR3* (10 CpGs) (Additional file 1: Table S10). Previously, *FOXP2* and *TACR3* have been linked to PAE-associated hypomethylated DMRs in mouse hippocampus [56] and rat prefrontal cortex [57], respectively. Furthermore, the DMP and DMR analyses brought forth several interesting genes such as *ANK3, CCDC3, WNT3, PALMD,* and *SEMA3B*, of which *PALMD* associated with PAE also in sensitivity analysis for DMPs (Additional file 1: Table S8). Regarding the retarded growth associated with FASD, previous associations between PAE and *IGF2/H19* locus [16, 58–60], and our earlier finding on the genotype-specific effects of PAE on DNAm of imprinting control region in *IGF2/H19* locus in the placenta [61], also DMR in *IGF2/IGF2AS* (21 CpGs) observed in the current genome-wide study with a larger sample size is highly interesting. This hypomethylated DMR spans 1756 bp and locates in the first exon of *IGF2* (Additional file 2: Fig. S7).

We also performed genome-wide DNAm analyses of placentas in the early PAE subgroup (*n* = 27). The analysis revealed 248 PAE-associated DMPs (170 hypomethylated and 78 hypermethylated, FDR < 0.05, Δ*β* ≤ −0.05 and Δ*β* ≥ 0.05) (Fig. 1b and Additional file 1: Table S11 as well as Tables S12 and S13 for sensitivity analyses), including hypomethylated DMPs in *DPPA4* and *TACR3* (two and six DMPs, respectively). Interestingly, two genes, *A2BP1* (also known as *RBFOX1)* and *DIP2C*, of which DMPs were observed in the early PAE placentas, have also been associated with altered DNAm in WBCs of early PAE newborns in previous meta-analysis of six population-based birth cohorts (*P*-values were not significant after multiple testing correction in this study) [62]. In both placenta and WBCs, there were hypermethylated DMP/CpG in the regulatory region of *A2BP1*, and hypomethylated DMP/CpG at the gene body of *DIP2C*—although not the same probes. Both genes have been associated previously with neuronal development and autism spectrum disorders (ASD) [63, 64]. DMR analysis for the early PAE subgroup revealed 29 DMRs, including *DPPA4* (4 CpGs) and *TACR3* (10 CpGs) (Additional file 1: Table S14).

Previously, PAE has been associated with altered cellular composition in human term placenta [65]. Therefore, although we study developmentally early alterations, which are expected to be present in subsequent cell lines derived from early developmental cell types exposed to PAE and consequently could be used as biomarkers, we excluded a potential bias in the results caused by cellular composition by cell-type-specific adjustment. Significantly increased proportions of trophoblast cells observed in all PAE placentas as well as in the early PAE subgroup compared to controls (*P* = 0.006, *P* = 0.038, respectively, Wilcoxon test) (Additional file 2: Fig. S8) are consistent with the PAE-associated increased number of villous cytotrophoblastic cells observed in the previous study [65]. Furthermore, the number of stromal cells was significantly lower in both groups of PAE placentas (*P* < 0.001, *P* < 0.001, respectively, Student’s *t* test) (Additional file 2: Fig. S8). However, the results of cell-type-specific DNAm analyses were consistent with the bulk results and confirmed the significance of the candidate genes *DPPA4*, *FOXP2*, and *TACR3* (Additional file 1: Tables S15–18).

Prominent PAE-associated hypomethylation of DMPs was observed in the majority of genomic locations relative to gene and CpG island in all PAE placentas—especially in the regulatory regions of placentas in the early PAE subgroup (Fig. 1c). In all PAE placentas, in relation to gene or CpG island, DMPs were enriched at the open sea (64.0% of the DMPs vs 56.7% in the EPIC array) and under-represented in the CpG island (9.3% vs 17.5%). In the subgroup of early PAE placentas, DMPs were enriched at the first exon (2.8% vs 1.0%) and south shore (16.1% vs 8.7%), and under-represented in CpG island (8.1% vs 17.5%) (*P* < 0.0001, *P* < 0.0001, *P* = 0.015, *P* = 0.0002, *P* < 0.0001, respectively, Fisher’s exact test followed by pairwise comparisons). The effect of PAE on genome-wide placental DNAm level was calculated by using 588,781 probes in the array, and significantly lower overall GWAM was observed in all PAE placentas compared to controls (*P* = 0.012, Student’s *t* test). This hypomethylation was seen in all genomic locations in both all placentas and the early PAE subgroup (Additional file 2: Fig. S9). The global placental DNAm level was also predicted by comparing the mean DNAm level of CpGs in Alu, LINE1, and LTR repetitive element regions, which comprise 36% of human genome in total [38, 66]. On the contrary to the hypomethylation observed when using DMPs or GWAM, significant hypermethylation was observed at LINE1 and LTR regions in all PAE placentas (*P* = 0.019 and *P* = 0.02, respectively, Student’s *t* test) and in LINE1s in the early PAE subgroup (*P* = 0.029, Student’s *t* test) compared with controls (Fig. 1d).

**Fig. 2:**
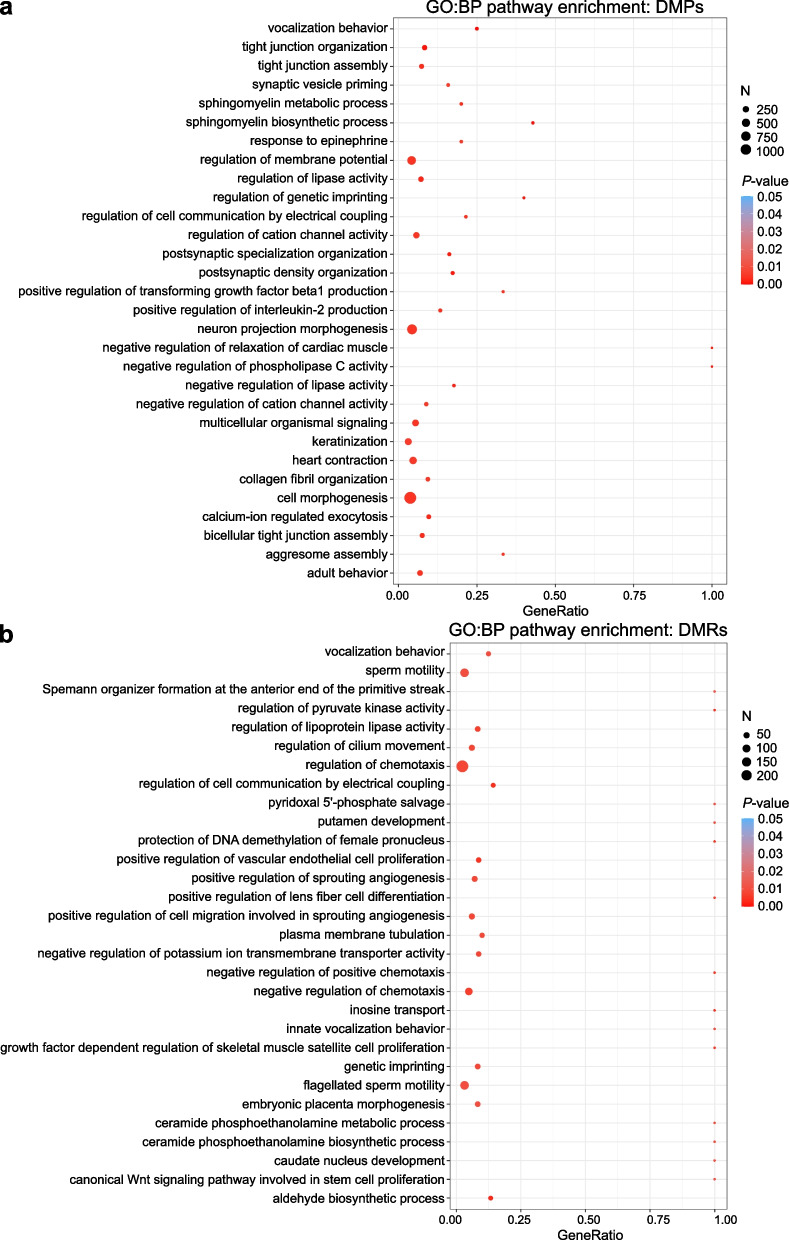
Pathway analyses of placental DMPs and DMRs. Enriched terms identified in GO:BP enrichment analysis of PAE-associated **a** DMPs and **b** DMRs in placenta (*P* < 0.05). In both figures the 30 most significant pathways are shown

Pathway analyses were performed to get a comprehensive picture on the biological processes in which PAE-associated DMPs cluster. GO enrichment analysis of PAE-associated DMPs revealed interesting BPs involved in the function of heart and nervous system as well as adult behavior (*P* < 0.05) (Fig. 2a and Additional file 1: Table S19). DMRs cluster to various BPs, such as the regulation of chemotaxis, embryonic placenta morphogenesis, Wnt signaling in stem cell proliferation, and putamen and caudate nucleus development (*P* < 0.05) (Fig. 2b and Additional file 1: Table S20). Both DMPs and DMRs cluster to the GO terms involved in vocalization behavior and genomic imprinting.

### Potential biomarkers for PAE

**Fig. 3:**
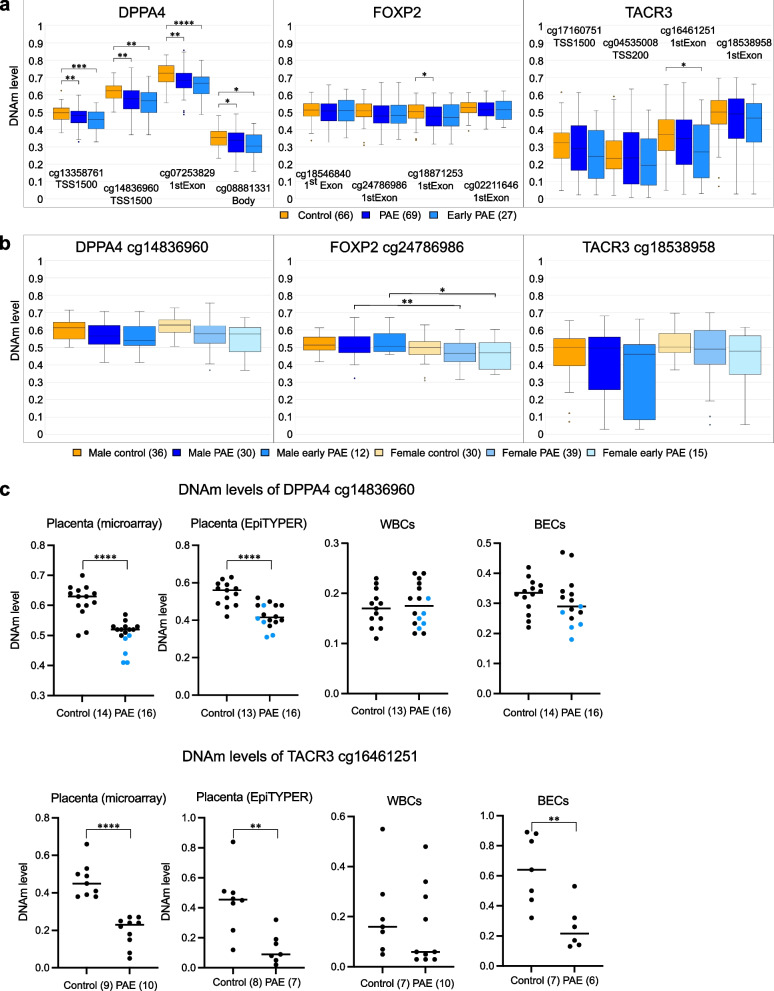
DNAm levels of potential PAE biomarkers *DPPA4, FOXP2,* and *TACR3*. **a** Box plots showing the normalized DNAm levels of DMPs and their locations in relation to gene in *DPPA4*, *FOXP2*, and *TACR3* in control, PAE, and early PAE placentas. Four DMPs with the largest effect sizes (mean Δβ) based on linear model of each candidate gene are presented. *P < 0.05, **P < 0.01, ***P < 0.001, ****P < 0.0001, two-tailed Student’s *t* test. **b** Box plots showing sex-specific normalized DNAm levels of DMPs with the largest effect sizes. *P < 0.05, **P < 0.01, two-tailed Student’s *t* test. **c** DNAm levels of *DPPA4* DMP (cg14836960) and *TACR3* DMP (cg16461251) in selected placental samples analyzed by microarrays and EpiTYPER as well as the same CpGs in WBCs and in BECs analyzed by EpiTYPER. Blue dots in *DPPA4* indicate DMP of each PAE placenta, which have the lowest DNAm levels in microarrays. **P < 0.01, ****P < 0.0001, Mann-Whitney *U*

To validate the results of genome-wide DNAm microarray analysis and to determine potential biomarkers for PAE, we examined DNAm profiles of *DPPA4, FOXP2,* and *TACR3* in placenta, WBCs, and BECs from each newborn by targeted DNAm analysis using EpiTYPER (Agena Bioscience, Inc.). Targets for the EpiTYPER analysis were selected based on the microarrays and the significance of the identified DMPs when all PAE placentas were compared to controls. Noteworthily, the normalized (not adjusted for smoking and sex) sample-specific DNAm levels were used in this analysis. There was a significant difference in the DNAm levels of *DPPA4* between control and all PAE placentas (cg13358761: *P* = 0.009, cg14836960: *P* = 0.002, and cg07253829: *P* = 0.003, cg08881331: *P* = 0.042, Student’s *t* test) (Fig. 3a). Interestingly, the difference was even more significant in the early PAE subgroup, which suggests early origin of these changes (cg13358761: *P* < 0.001, cg14836960: *P* = 0.001, and cg07253829: *P* < 0.0001, cg08881331: *P* = 0.015, Student’s *t* test). Although some significant differences in the normalized DNAm levels of *FOXP2* or *TACR3* were detected (cg18871253 (all PAE placentas): *P* = 0.047, and cg16461251 (early PAE subgroup): *P* = 0.047, respectively) the differences were smaller than expected. Considering the significance of these genes in the sensitivity analyses without smoking-exposed samples (adjusted only for sex), we tested the effect of sex on the DNAm levels of the DMPs with the largest effect size (Fig. 3b). Interestingly, when we compared DNAm levels between males and females, we observed significant sex-specific effects in *FOXP2* in both all PAE placentas and in the early PAE subgroup (*P* = 0.01*, P* = 0.023, respectively, Student’s *t* test).

The placental samples with the largest DNAm difference between PAE and control groups were chosen for the further analysis (*DPPA4* (cg14836960) and *TACR3* (cg16461251) *P* < 0.001, *FOXP2* (with two DMPs in the same unit: cg18871253 and cg24786986) *P* = 0.002, Mann-Whitney *U*, Additional file 1: Table S21, selected samples in the Additional file 1: Tables S1 and S2). The DNAm levels of selected DMPs in placenta measured by the microarrays and EpiTYPER correlated significantly, and thus validated the DNAm results as well as the feasibility of the selected EpiTYPER primers (*DPPA4*: *r* = 0.856, *P* = 3.29×10^−9^, *n* = 29; *FOXP2*: *r* = 0.962, *P* = 4.72×10^−11^, *n* = 19; and *TACR3*: *r* = 0.903, *P* = 4.06×10^−6^, *n* = 15, respectively, Spearman’s rank correlation).

We next examined the applicability of observed PAE-associated placental DNAm differences for potential PAE biomarkers in blood or BECs, which are more easily accessible biological samples than placenta. The stability of DNAm levels across placenta, WBCs, and BECs from each of the selected newborns was tested, but significant correlations between tissues or cell types were not observed. However, when only the PAE newborns with the lowest placental DNAm level of *DPPA4* DMP (cg14836960) were scrutinized, a trend of low DNAm level was detected also in BECs of the same newborns (Fig. 3c). Furthermore, a trend of PAE-associated hypomethylation of DMP in the first exon of *TACR3* (cg16461251) was observed also in WBCs and BECs (Fig. 3c). Notably, the DNAm difference in this specific DMP between selected control and PAE placentas was also significant in BECs analyzed by EpiTYPER (*P* = 0.009, Mann-Whitney *U*).

### Effects of PAE on placental mRNA expression

**Fig. 4:**
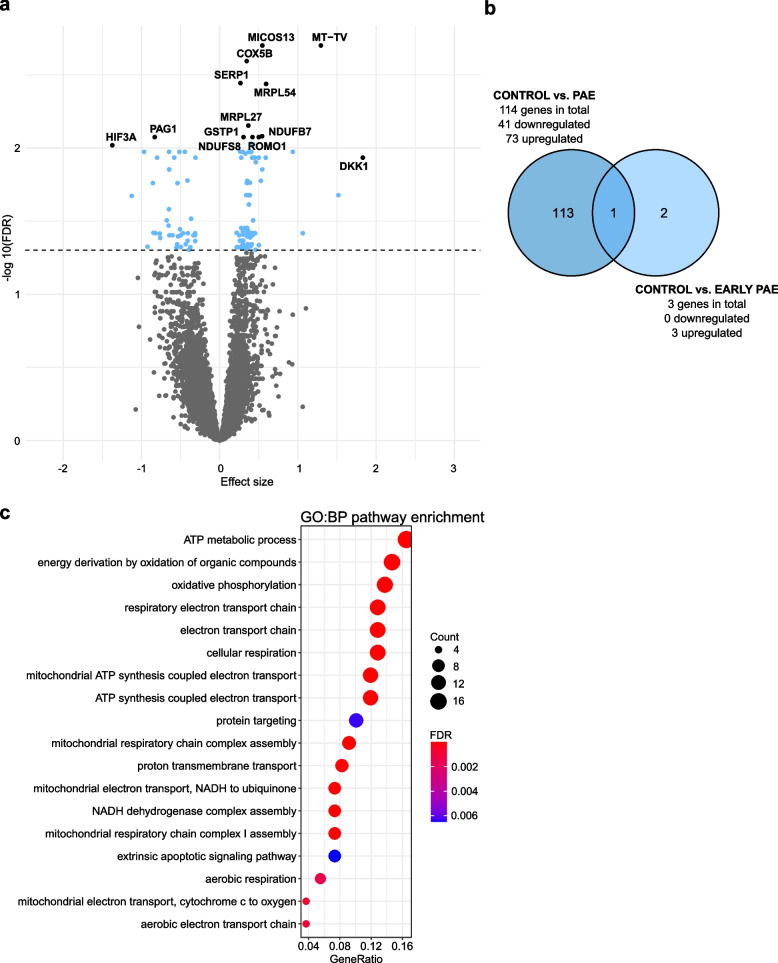
PAE-associated differential gene expression in the placenta. **a** Volcano plot showing the distribution of associations between mRNA expression and PAE. Horizontal line marks FDR 0.05. **b** Venn diagram showing the number of PAE-associated differentially expressed genes, which are in common between all PAE placentas and the early PAE subgroup. **c** Significantly enriched terms identified in GO:BP enrichment analysis of PAE-associated differentially expressed genes in placenta (FDR-corrected *q*-value < 0.05). The 30 most significant pathways are shown. Control *n* = 41, PAE *n* = 64, and early PAE *n* = 23

To study genome-wide PAE-associated alterations in gene expression, we performed mRNA-seq for 64 PAE and 41 control placentas (Samples in Additional file 1: Tables S1 and S2, and general characteristics in Additional file 1: Table S3). When the mRNA-seq model was adjusted by smoking and sex, we observed 114 significantly differentially expressed genes (FDR < 0.05) of which 41 were downregulated and 73 upregulated (Fig. 4a,b and Additional file 1: Table S22). According to the GO:BP enrichment analysis, PAE-associated gene expression is linked predominantly to cellular respiration in mitochondria (FDR-corrected *q*-value < 0.05) (Fig. 4c and Additional file 1: Table S23). Indeed, the majority of the most significantly differentially expressed genes (FDR < 0.01) have roles in mitochondrial function and cellular respiration (*MICOS13*, *MT-TV*, *COX5B*, *SERP1*, *MRPL54*, *MRPL27*, *NDUFB7*, *GSTP1*, and *NDUFS8*), in increasing the level of reactive oxygen species (ROS) (*ROMO1*) [67] as well as in cellular response to oxidative stress (*PAG1*) [68] and hypoxia (*HIF3A*) [69]. This is consistent with previous studies, since alcohol exposure has been associated with increased oxidative stress and mitochondrial dysfunction in several tissues [70, 71], including rat placenta [72]. Increased oxidative stress is characteristic for several gestational disorders such as intrauterine growth restriction and preterm birth [73, 74], which are also known characteristics of PAE. According to our analysis, *DPPA2*, *DPPA4*, *FOXP2*, or *TACR3* are expressed below the detectable level in the term placenta.

Three genes, *DKK1*, *RBP4*, and *UCHL1*, were significantly upregulated in the placentas of the early PAE subgroup (*n* = 23, FDR < 0.05) (Additional file 1: Table S24). *Dickkopf1 (DKK1)*, which was significantly upregulated also in all PAE placentas (Fig. 4a,b), is crucial for head, limb, and heart development [75, 76]. It is an inhibitor of the Wnt/β-catenin signaling pathway [77], which among various developmental processes has an essential role in the development of early trophoblasts [78]. Interestingly, increased expression of *DKK1* has been associated previously with pre-eclampsia [79, 80] and unexplained recurrent spontaneous miscarriage [81].

To characterize the potential effect of PAE-associated DNAm on gene expression levels in the same samples, we performed correlation analysis. The analysis revealed nine genes, which had significant PAE-associated correlation between decreased DNAm and increased mRNA expression in the placenta: *B3GNT3, CBR1, CNDP2, HEATR5A, PRKAG2, S100A14, SAR1B, STEAP3,* and *TUSC3* (*P* < 0.01) (Additional file 1: Table S25). All the genes, except *B3GNT3* and *PRKAG2*, have correlative probes in the regulatory regions*.*

### Effects of in vitro alcohol exposure on hESCs

**Fig. 5:**
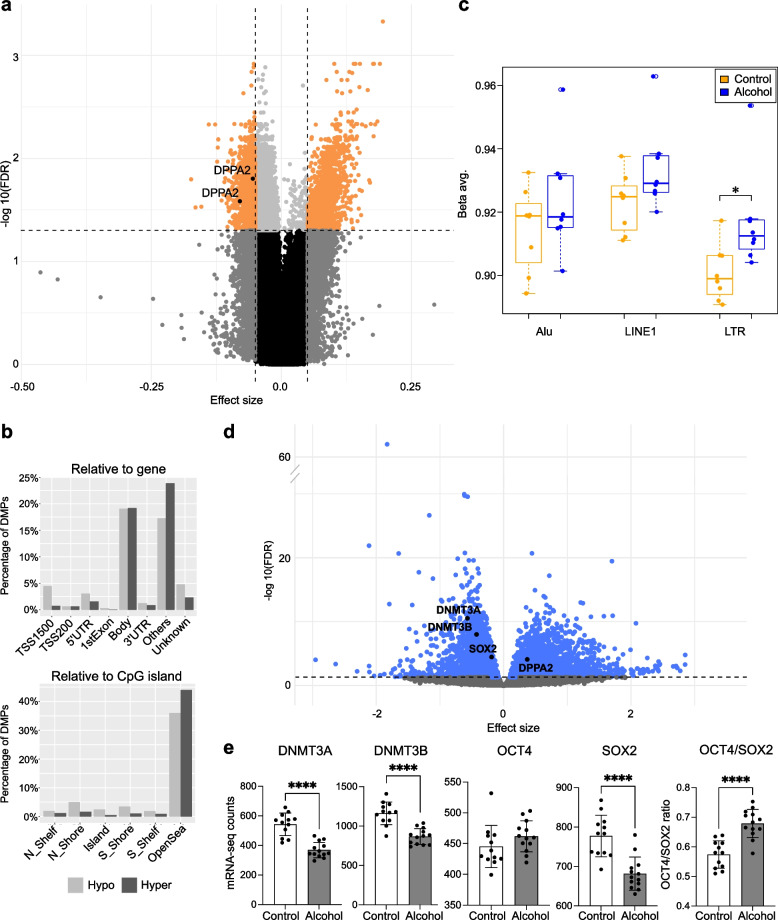
Effects of alcohol exposure on hESCs. **a** Volcano plot showing the distribution of associations between CpG sites and alcohol exposure in hESCs. Horizontal line marks FDR 0.05 and vertical line marks effect size ± 0.05. **b** Location of alcohol-induced DMPs in relation to gene and CpG island in hESCs. DMPs were divided to hypo- and hypermethylated subgroups, which were further grouped according to the genomic location based on UCSC database. If the location information was missing, DMP was marked as “unknown.” In the case of multiple location entries, group “others” was used. **c** Effects of alcohol exposure on global DNAm level in hESCs predicted by Alu, LINE1, and LTR repetitive regions. *P < 0.05, two-tailed Student’s *t* test. **d** Volcano plot showing the distribution of associations between mRNA expression and alcohol exposure. Horizontal line marks FDR 0.05. **e***DNMT3A*, *DNMT3B, OCT4*, and *SOX2* gene expressions as well as *OCT4*/*SOX2* expression ratio in control and alcohol-exposed hESCs. Data presented as mean ±SD. *P < 0.05, two-tailed Student’s *t* test and ****P < 0.0001, FDR-corrected *P*-value. Control hESCs *n* = 8 and *n* = 12 as well as alcohol-exposed hESCs *n* = 8 and *n* = 13 in DNAm and mRNA-seq analyses, respectively. Abbreviations TSS1500: 1500 bp upstream of transcription start site, TSS200: 200 bp upstream of TSS, UTR: untranslated region, N_shelf: north shelf, N_shore: north shore, S_shore: south shore, S_shelf: south shelf

To explore the effects of early alcohol exposure on hESCs without potential confounding factors associated with human studies in vivo, we exposed two cell lines (H1 and Regea08/017) with replicates to 70 mM alcohol for 48 h and performed genome-wide DNAm and gene expression analyses. By using Illumina’s EPIC microarrays (eight alcohol-exposed and eight control samples), we identified 10,888 alcohol-induced differentially methylated CpG sites (10,046 hypomethylated and 842 hypermethylated) as well as 1111 hypermethylated non-CpG sites (mCpHs) common in hESCs with FDR < 0.05 (Fig. 5a and Additional file 1: Table S26). Of all differentially methylated sites, 3700 (1879 hypomethylated CpGs, 714 hypermethylated CpGs, and 1107 hypermethylated CpHs) were considered as significant DMPs (FDR < 0.05, Δ*β* ≤ −0.05 and Δ*β* ≥ 0.05) and were used in further analysis. Furthermore, a total of 442 DMRs were observed (Additional file 1: Table S27).

The global trends of alcohol-associated DNAm changes were consistent between in vivo alcohol-exposed placentas and in vitro exposed hESCs. GWAM analysis revealed significant alcohol-induced genome-wide hypomethylation (*P* = 0.005, Student’s *t* test), which was seen in all genomic locations (Additional file 2: Fig. S10). Prominent hypomethylation was also observed in most genomic locations relative to gene and CpG island when only DMPs were analyzed (Fig. 5b). DMPs were enriched at the gene body (38.2% of the DMPs vs 34.8% in the EPIC array) and open sea (80.0% vs 56.4%), and under-represented in the TSS1500 (5.2% vs 9.1%), TSS200 (1.3% vs 6.1%), first exon (0.3% vs 1.0%), north shore (6.7% vs 9.7%), CpG island (2.9% vs 18.7%), south shore (4.5% vs 8.2%), and south shelf (2.8% vs 3.4%) (*P* < 0.0001, *P* < 0.0001, *P* < 0.0001, *P* < 0.0001, *P* < 0.0001, *P* < 0.0001, *P* < 0.0001, *P* < 0.0001, *P* = 0.04, respectively, Fisher’s exact test followed by pairwise comparisons). The global DNAm level predicted by Alu, LINE1, and LTR repetitive element regions was significantly higher in LTRs of alcohol-exposed hESCs (*P* = 0.02, Student’s *t* test) (Fig. 5c). Also, according to the GO:BP enrichment analysis, DMPs and DMRs of alcohol-exposed hESCs cluster to the GO terms involved in the function of heart and nervous system, consistently with PAE placentas (*P* < 0.05) (Additional file 1: Tables S28 and S29, Additional file 2: Fig. S11 and S12, respectively).

To study alcohol-induced alterations in gene expression, we performed mRNA-seq analysis for the same two hESC lines with replicates (13 alcohol-exposed and 12 control samples). A total of 4992 genes with significantly altered expressions were observed (FDR < 0.05) (Fig. 5d and Additional file 1: Table S30), which are predominantly linked to the RNA processing and mitochondrial gene regulation according to the GO:BP enrichment analysis (FDR-corrected *q*-value < 0.05) (Additional file 1: Table S31 and Additional file 2: Fig. S13). The correlation analysis revealed two genes (*DPEP3, RAB17*), which had a significant PAE-associated correlation between increased DNAm and decreased mRNA expression in hESCs (Additional file 1: Table S32).

The expression of developmentally critical genes, pluripotency gene *SOX2*, and both de novo DNA methyltransferase enzymes *DNMT3A* and *DNMT3B* (FDR = 3.52×10^−5^, FDR = 3.08×10^−11^ and FDR = 1.02×10^−8^, respectively) was significantly downregulated in alcohol-exposed hESCs (Fig. 5e). Furthermore, the ratio of *OCT4* to *SOX2* was significantly higher in alcohol-exposed hESCs compared to controls (*P* = 1.18×10^−5^, Student’s *t* test) (Fig. 5e), which is consistent with a previous study with mouse ESCs [8]. Interestingly, it has been shown that the differentiation into the germ layers in mouse depends on the dosage of pluripotency genes *Oct4* and *Sox2* and that *Sox2* protein level is upregulated in cells differentiating into neural ectoderm [82].

### Effects of early in vitro alcohol exposure on DPPA2 and DPPA4 regulation

**Fig. 6:**
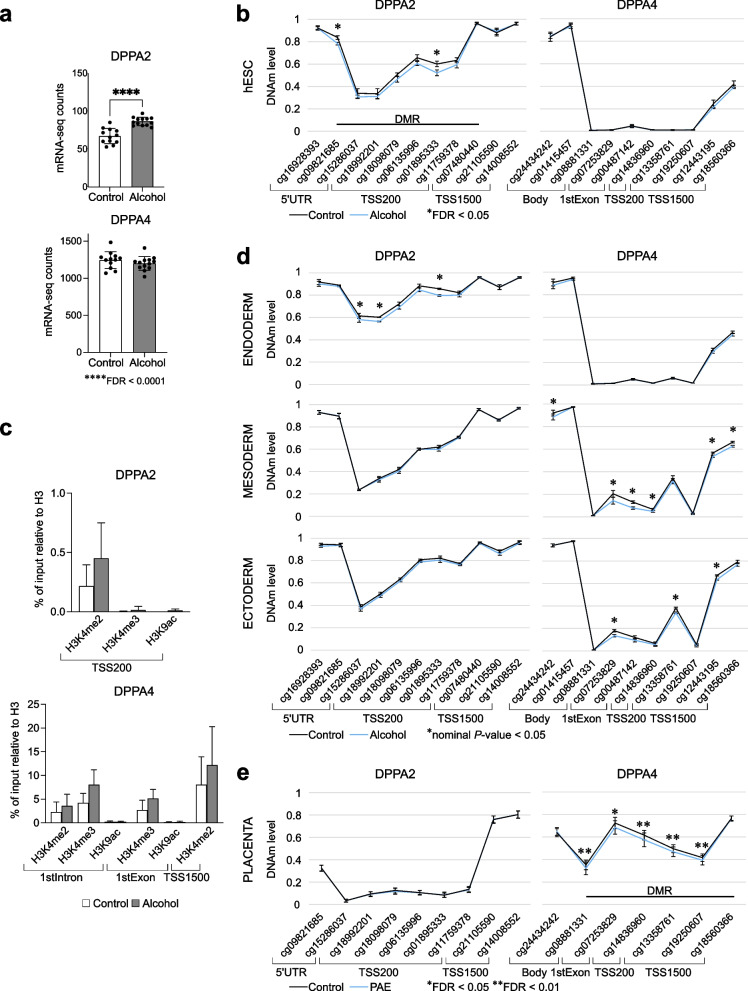
Effects of alcohol exposure on *DPPA2* and *DPPA4* in hESCs and differentiating cells. **a***DPPA2* and *DPPA4* gene expressions in control (*n* = 12) and alcohol-exposed (*n* = 13) hESCs. **b***DPPA2* and *DPPA4* DNAm profiles in control and alcohol-exposed hESCs (*n* = 8/condition). **c***DPPA2* and *DPPA4* histone modifications in control and alcohol-exposed hESCs (*n* = 4/condition). Histone modification enrichments were normalized to the total histone H3. **d***DPPA2* and *DPPA4* DNAm profiles in control and alcohol-exposed differentiated endo-, meso-, and ectodermal cells (*n* = 4/condition, respectively). **e***DPPA2* and *DPPA4* DNAm profiles in control (*n* = 66) and PAE (*n* = 69) placentas. Data presented as mean ±SD. *P < 0.05, Mann-Whitney *U* and ****P < 0.0001, FDR-corrected *P*-value

Owing to the function of *DPPA2* and *DPPA4* in the early embryonic development, we explored if alcohol exposure could affect their DNAm and expression in hESCs or after in vitro differentiation into the three germ layers. The genome-wide expression analysis for hESCs revealed significant alcohol-induced upregulation of *DPPA2* (FDR *=* 8.36×10^−5^), but no change in considerably actively expressed *DPPA4* (Fig. 6a). This is in line with the results of genome-wide DNAm analysis—there were two hypomethylated DMPs and one DMR in the regulatory region of *DPPA2*, but the regulatory region of *DPPA4* was unmethylated in both alcohol-exposed and control hESCs (Fig. 5a and b, Fig. 6b) (confirmed by EpiTYPER analysis, Additional file 1: Table S33). Furthermore, we studied potential alcohol-induced alterations in histone modifications H3K4me2, H3K4me3, and H3K9ac by ChIP-qPCR. The trends of the observed changes were consistent with the alcohol-induced alterations in DNAm and gene expression, but they were not statistically significant. Although the level of histone modifications in the regulatory region of *DPPA2* was very low compared to *DPPA4*, we observed increased amount of active chromatin mark H3K4me2 in the regulatory regions of both *DPPA2* and *DPPA4* as well as increased amount of H3K4me3 in *DPPA4* in alcohol-exposed hESCs (Fig. 6c).

To see the potential effects of alcohol exposure on DNAm profiles of *DPPA2* and *DPPA4* regulatory regions in differentiating cells, we differentiated hESCs (H1) into the endodermal, mesodermal, and ectodermal cells in vitro. The cells were exposed to 70 mM alcohol during the culturing and the DNAm profiles of *DPPA2* and *DPPA4* loci were analyzed from normalized Illumina’s EPIC microarray data (four alcohol-exposed and four control samples/germ layer). The DNAm profile of endodermal cells was similar to hESCs with significant locus-specific decreased DNAm in *DPPA2* regulatory region in alcohol-exposed cells and unmethylated *DPPA4* regulatory region in both alcohol-exposed and control cells (Fig. 6d). On the contrary to the hESCs, increased DNAm level in the *DPPA4* regulatory region was observed in mesodermal and ectodermal cells. Notably, consistent with the PAE placentas, both mesodermal and ectodermal cells had significant locus-specific alcohol-induced decreased DNAm in the regulatory region of *DPPA4* (Fig. 6d,e).

### Genome-wide effects of in vivo and in vitro alcohol exposure

**Fig. 7:**
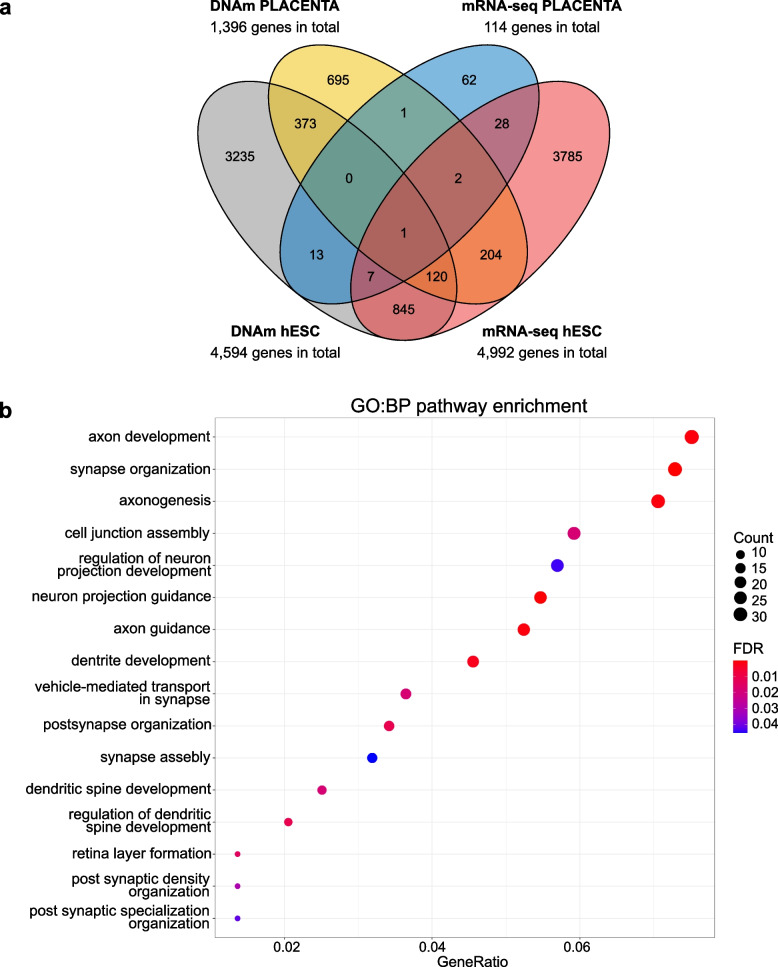
Common alcohol exposure-associated genes in the genome-wide analyses. **a** Venn diagram showing the number of common genes, which associate with alcohol exposure in the genome-wide DNAm and mRNA-seq analyses of placenta and hESCs. **b** Significantly enriched terms identified in GO:BP enrichment analysis of 494 common genes in DNAm analyses of placenta and hESCs (FDR-corrected *q*-value < 0.05). All the significant pathways are shown

Finally, to see the early effects of alcohol exposure on genes in both in vivo exposed extraembryonic placenta and in vitro exposed hESCs, we compared the results of genome-wide DNAm and mRNA-seq analyses (Fig. 7a and Additional file 1: Table S34). The comparisons were performed by using all significant CpGs (FDR < 0.05) and significantly differentially expressed genes (FDR < 0.05). Only one common gene, *TBC1 domain family member 5* (*TBC1D5*), associated with alcohol exposure in all genome-wide analyses of placenta and hESCs. *TBC1D5* encodes a GTPase-activating protein involved in hepatic lipophagy [83], which protects the liver from alcohol-induced fatty liver disease [84]. It has been shown to be downregulated by chronic alcohol administration in mouse liver and ethanol-treated HepG2 cells [85]. In the current study, *TBC1D5* was downregulated in PAE placentas (one hypomethylated CpG in 5’UTR) but upregulated in alcohol-exposed hESCs (two hypomethylated CpGs in the gene body).

A total of 494 common genes associated significantly with alcohol exposure in DNAm analyses of placenta and hESCs. Notably, according to the GO:BP enrichment analysis, these common genes are linked exclusively to neurodevelopmental GO terms including axon development and synapse organization (FDR-corrected *q*-value < 0.05) (Fig. 7b and Additional file 1: Table S35). Importantly, several of these common genes have been previously associated with PAE or FASD in BECs or/and peripheral WBCs in children (Additional file 1: Table S36). *PTPRN2* [86], *MAD1L1* [87], and *AGAP1* [88] are all linked to neurodevelopmental disorders and associate significantly with PAE or FASD in two or more previous genome-wide DNAm studies [16, 17, 62, 89]. Furthermore, *FOXP1*, a family member of *FOXP2*, and *GLI2* have been found to associate with FASD diagnosis in BECs [89] or WBCs [15] in childhood, respectively. Interestingly, transcription factor *GLI2* is a mediator of Sonic hedgehog signaling, and it has been earlier associated with facial dysmorphology and brain deficiency in alcohol-exposed mouse fetuses [90].

### Associations between candidate genes, alcohol consumption, and newborns’ phenotypes

Potential correlations between placental DNAm and gene expression of the candidate genes and maternal alcohol consumption were calculated. DMPs with the largest effect sizes were examined. A moderate negative correlation between *TACR3* DNAm and ad was observed in the all PAE group (cg16461251: *r* = −0,403, *P* = 0.041 and cg18538958: *r* = −0.395, *P* = 0.046, *n* = 26, Spearman’s rank correlation) and a strong negative correlation in the early PAE subgroup (cg18538958: *r* = −0.762, *P* = 0.028, *n* = 8, Spearman’s rank correlation). When the correlations between placental DNAm and newborns’ birth measures (SDs) and placental weight (g) were examined, no correlations in the all PAE group were detected. However, in the early PAE subgroup, *DPPA4* and *FOXP2* DNAm correlated moderately and negatively with the birth weight (cg14836960: *r* = −0,431, *P* = 0.025, cg18546840: *r* = −0.415, *P* = 0.032, respectively, *n* = 27, Pearson correlation). Also, *FOXP2* DNAm correlated moderately and negatively with the birth length (*r* = −0.466, *P* = 0.014, Pearson correlation). Interestingly, the *DPPA4* DMP (cg14836960) correlated significantly with *FOXP2* (cg18546840: *r* = 0.491, *P* = 0.009, cg24786986: *r* = 0.466, *P* = 0.0014, respectively, *n* = 27, Pearson correlation) and *TACR3* (cg16461251: *r* = 0.415, *P* = 0.031, *n* = 27, Pearson correlation) DMPs in the early PAE subgroup, but no correlations were observed in the all PAE placentas group. Also, the potential effect of alcohol consumption on genome-wide DNAm was calculated, but no correlation between GWAM and AUDIT scores or ad were found.

## Discussion

Our study, which is the first genome-wide DNAm analysis of severely alcohol-exposed placentas as far as we are aware, strengthens the value of placental tissue in studying the effects of prenatal environment on human development. This can be seen in similar locus-specific DNAm alterations in *DPPA4* and *TACR3* in alcohol-exposed extraembryonic and embryonic cells as well as in similar global DNAm changes in both in vivo and in vitro exposed cell types. Also, the common genes with alcohol-associated DNAm changes in placenta and hESCs were linked to the neurodevelopmental pathways. Alterations in genes involved in axon development or synapse organization may not be essential for placental cells, but DNAm changes can reveal the effects of early environment on epigenome in general, without cell or tissue specificity.

The role of *DPPA2* and *DPPA4* as chromatin modifiers and epigenetic priming factors in early development makes them plausible candidate genes for developmental disorders. Alcohol-induced alteration in the regulation of *DPPA2* in hESCs as well as decreased DNAm in *DPPA4* regulatory region in PAE placentas and alcohol-exposed mesodermal and ectodermal cells indicates that alcohol is able to affect their regulation in the early development. Delayed downregulation of epigenetic priming factors during a critical period of development can result in subtle but widespread alterations in the timing and efficiency of developmental programming. Indeed*,* increased levels of both *DPPA2* and *DPPA4* have enhanced reprogramming to pluripotency in mouse and human cells [11] and expression of *DPPA4* has been associated with inhibition of ESC differentiation into a primitive ectoderm lineage in mouse [91]. Furthermore, our study shows that alcohol exposure alters the balance of *OCT4* and *SOX2* expression in hESCs, which is in line with a previous mouse study [8]. Owing to the effects of these two lineage specifier proteins on the differentiation into mesoendoderm or neuronal ectoderm [82, 92, 93], our results are consistent with the idea that alcohol can affect the cell fate decision and consequently reduce the number of ectodermal cells [8]. Observed changes in *DPPA4, OCT4*, and *SOX2* could explain the specific vulnerability of the developing nervous system to the effects of alcohol.

Another candidate gene for alcohol-induced developmental disorders identified in the current study is the transcription factor *FOXP2*, which represses genes involved in maintaining a non-neuronal state and activates genes that promote neuronal maturation by affecting chromatin structure [94]. Neuronal phenotypes associated with *FOXP2* mutations include expressive and receptive language impairment, orofacial dyspraxia, abnormalities in cortex, and basal ganglia [53, 95, 96] as well as attention deficit hyperactivity disorder (ADHD) [97]. Since receptive and expressive language disorders as well as ADHD have been considered common comorbidities of FASD [98, 99], *FOXP2* is a plausible candidate gene for developmental disorders induced by PAE. Interestingly, both *FOXP1* and *FOXP2* as well as their targets have been associated with ASD [100–102], which could explain the partial overlapping phenotypes between FASD, ASD, and ADHD observed in previous studies [98, 103].

The observed difference in *DPPA4* DNAm between PAE and control placentas is a potential biomarker for early alcohol exposure or even FASD, but the use of placental tissue in diagnostics has limitations. The PAE-associated trend of decreased DNAm in *TACR3* in both newborns’ placenta and BECs however suggests that alterations are detectable in different tissue types with variable cell-type compositions, which makes buccal swabs a promising tool for diagnostics. Due to the ectodermal origin of BECs, buccal samples could be particularly useful for the diagnostics of neurodevelopmental disorders. However, when placental DMPs were compared to all four previous genome-wide DNAm studies of BECs [16, 17, 89, 104], no common PAE- or FASD-associated probes or genes were found (Additional file 1: Table S37). Although a total of 43 genes in the current study associated with PAE or FASD in some of the previous studies, our three candidate genes, *DPPA4, FOXP2*, or *TACR3*, have not been detected earlier. The inconsistency could be explained by the cellular heterogeneity, different age of the affected children, or differences in genetic background. Owing to *TACR3*’s role in growth, reproduction, and several processes in the nervous system [105, 106] as well as the moderate correlation between placental *TACR3* hypomethylation and alcohol units consumed per week, it is also an interesting candidate gene for alcohol-induced developmental disorders. Since genetic variation in *TACR3* has been associated with alcohol dependence [54], potential genotypic effects on observed PAE-associated hypomethylation should be studied in the future. Furthermore, it has been shown previously [107, 108] as well as in this study that PAE has sex-specific effects. Although it was not in the focus of the current study, it requires further investigation.

The majority of the alcohol-associated DMPs in the regulatory regions in both placenta and hESCs were hypomethylated, and also significant alcohol-associated genome-wide hypomethylation in all genomic locations based on GWAM was observed. On the contrary, by using repetitive elements, we predicted increased global alcohol-associated DNAm in both placenta and hESCs. This is consistent with the previous study, in which PAE throughout the pregnancy has been associated with higher placental global DNAm examined by using Alu repeats of male newborns [109]. Also, the increased DNAm at the LTR promoter of intracisternal A particle in the *agouti* locus was observed in our previous study, in which we showed for the first time that PAE can affect adult phenotype by altering the epigenotype of early mouse embryo [5]. The mechanisms by which alcohol alters the DNAm are still mainly unknown. Enzymatic malfunction of DNMTs caused by oxidative stress or effects of alcohol on cells’ methionine cycle and consequently on DNAm level have been suggested in previous studies [110]. Also, according to previous studies, the timing of exposure is fundamental—the effects differ between undifferentiated, differentiating, and differentiated cells [7, 111].

We explored more specifically the effects of early PAE by selecting 27 newborns whose mothers had consumed alcohol up to GW 7 at maximum to the separate analyses. Observed similar trends of PAE-associated DNAm alterations in all PAE placentas as well as in the early PAE subgroup suggest that there are changes that have occurred already in the early pregnancy. Also, the significant correlation of *DPPA4* DNAm with *FOXP2* and *TACR3* only in the early PAE subgroup suggests a parallel effect of early exposure on these three candidate genes in each placenta, which may be confounded during prolonged exposure. Although the birth weight (SD) or length (SD) of the newborns in this subgroup did not differ from controls, their significantly smaller HC (SD) suggests adverse effects of early PAE on brain development. Due to the increasing alcohol consumption among women in childbearing age [112] and a large proportion (44–65%) of unplanned pregnancies [113], there is a considerable risk of PAE and consequently neurodevelopmental disorders prior to pregnancy recognition. This developmentally critical period, the first weeks of pregnancy from fertilization to gastrulation and to the beginning of organogenesis, should be in the focus of future studies. Especially the role of Wnt signaling in the etiology of FASD should be explored, since this developmentally crucial pathway brought forth in several of our analyses.

We are aware of the limitations in this study. We have been able to focus only on gestational alcohol consumption, although the effects of parental alcohol consumption on gametes prior to fertilization can also affect embryonic development [114, 115]. Also, the effects of common concurrent factors such as smoking, antidepressants, and other drugs and their interactions, cannot be completely excluded. Although we managed to separate PAE-specific alterations, the confounding effect of smoking can be seen in both DNAm and mRNA-seq analyses. The high number of placentas exposed to both alcohol and smoking (82.5%) as well as potential interaction of these two or other potential factors may explain the smaller effect size of PAE than was expected. Notably, when PAE-associated genes were compared to a meta-analysis of placental DNAm changes associated with maternal smoking [116], nine common genes were found. However, seven of these genes were detected also in the sensitivity analyses of the non-smoking samples, and according to this, only *INPP5A* and *MGRN1* associated with smoking in the current study. Finally, we need to consider that the amount and the timing of consumed alcohol is mainly self-reported by the mothers in a special outpatient clinic for pregnant women with substance use problems, and inaccuracy in this personal evaluation can occur [117]. Due to these limitations, in vitro experiments are a necessary part of this study.

## Conclusions

By using the unique biological samples of PAE newborns as well as alcohol-exposed both hESCs and differentiated hESCs, our study shows the early effects of alcohol exposure on both embryonic and extraembryonic cells reveal interesting new candidate genes *DPPA4*, *FOXP2*, and *TACR3* for the effects of PAE as well as brings forth potential biomarkers for PAE or even FASD. The discovery of *DPPA4* and *FOXP2* introduces the role of chromatin modifiers in alcohol-induced developmental disorders in human. Inaccurate timing and efficiency of transcriptional programming due to unfavorable epigenetic environment could explain the wide spectrum of disorders in the FASD phenotype.

## Supplementary Information

**Additional file 1: Table S1.** General information about PAE samples. **Table S2.** General information about control samples. **Table S3.** General characteristics of PAE, early PAE and control newborns as well as their mothers included in the genome-wide DNAm and mRNA-seq analyses. **Table S4.** Primers for EpiTYPER and ChIP-qPCR. **Table S5.** PCR protocols for EpiTYPER and ChIP-qPCR. **Table S6.** Antibodies for ChIP. **Table S7.** PAE-associated differentially methylated CpGs in the placenta analyzed by microarrays. **Table S8.** PAE-associated differentially methylated CpGs in the placenta: sensitivity analysis for DMPs. **Table S9.** PAE-associated differentially methylated CpGs in the placenta: sensitivity analysis for candidate genes. **Table S10.** PAE-associated DMRs in the placenta. **Table S11.** Early PAE-associated differentially methylated CpGs in the placenta. **Table S12.** Early PAE-associated differentially methylated CpGs in the placenta: sensitivity analysis for DMPs. **Table S13.** Early PAE-associated differentially methylated CpGs in the placenta: sensitivity analysis for candidate genes. **Table S14.** Early PAE-associated DMRs in the placenta. **Table S15.** PAE-associated differentially methylated CpGs in the placenta: adjusted by cell type. **Table S16.** PAE-associated DMRs in the placenta: adjusted by cell type. **Table S17.** Early PAE-associated differentially methylated CpGs in the placenta: adjusted by cell type. **Table S18.** Early PAE-associated DMRs in the placenta: adjusted by cell type. **Table S19.** Pathway analysis of placental DMPs. **Table S20.** Pathway analysis of placental DMRs. **Table S21.** Sample-specific normalized placental DNAm levels (analyzed by microarrays) of target genes selected for EpiTYPER analysis. **Table S22.** PAE-associated differentially expressed genes in the placenta analyzed by mRNA-seq. **Table S23.** Pathway analysis of differentially expressed genes in PAE placentas. **Table S24.** Early PAE-associated differentially expressed genes in the placenta. **Table S25.** Genes with significant PAE-associated correlation between placental DNAm and mRNA expression. **Table S26.** Alcohol-induced differentially methylated CpGs and mCpHs in hESCs analyzed by microarrays. **Table S27.** Alcohol-induced DMRs in hESCs. **Table S28.** Pathway analysis of DMPs in hESCs. **Table S29.** Pathway analysis of DMRs in hESCs. **Table S30.** Differentially expressed genes in alcohol-exposed hESCs analyzed by mRNA-seq. **Table S31.** Pathway analysis of differentially expressed genes in hESCs. **Table S32.** Genes with significant alcohol exposure-associated correlation between DNAm and mRNA expression in hESCs. **Table S33.** DNAm levels of *DPPA4* regulatory region in alcohol-exposed and control hESCs analyzed by EpiTYPER. **Table S34.** Common alcohol exposure-associated genes in genome-wide DNAm and mRNA-seq analyses of placenta and hESCs. **Table S35.** Pathway analysis of common genes associated significantly with alcohol exposure in genome-wide DNAm analyses of placenta and hESCs. **Table S36.** Common genes in genome-wide DNAm analyses of placenta and hESCs previously associated with alcohol exposure. **Table S37.** Common genes and DMPs in genome-wide DNAm analysis of placenta previously associated with alcohol exposure in BECs of FASD or PAE children.

**Additional file 2: Figure S1.** Timing and amount of maternal alcohol consumption in categories **Figure S2.** Characterization of hESC differentiation into germ layer cells **Figure S3.** Q-Q plots of genome-wide DNAm (adjusted by smoking and sex) and sensitivity analyses (adjusted by sex, maternal age, mode of delivery, and parity) **Figure S4.** SVD plots of sensitivity analysis for candidate genes **Figure S5.** PCA for PAE-associated DMPs in placenta **Figure S6.** Effect sizes of candidate gene DMPs in genome-wide DNAm and sensitivity analyses **Figure S7.** Schematic figure about PAE-associated DMR at *IGF2/H19* locus in placenta **Figure S8.** Cell-type composition in placental samples **Figure S9.** GWAM comparison between control and PAE placentas **Figure S10.** GWAM comparison between alcohol-exposed and control hESCs **Figure S11.** Pathway analysis of DMPs in hESCs **Figure S12.** Pathway analysis of DMRs in hESCs **Figure S13.** Pathway analysis of differentially expressed genes in hESCs.

## Data Availability

The datasets supporting the conclusions of the current study are included within the article and its additional files. Due to the sensitive nature of the patient data used, the data sets are not and cannot be made publicly available. The other data that support the findings of this study, such as the codes, is available from the corresponding author, NKA, upon reasonable request.

## Abbreviations

Ad: Alcohol units consumed per week
ADHD: Attention deficit hyperactivity disorder
ASD: Autism spectrum disorder
AUDIT: Alcohol Use Disorders Identification Test
BEC: Buccal epithelial cells
BP: Biological process
ChIP: Chromatin immunoprecipitation
CS: Caesarian section
DKK1: Dickkopf1
DMP: Differentially methylated position
DMR: Differentially methylated region
DNAm: DNA methylation
DPPA2: Developmental pluripotency associated factor 2
DPPA4: Developmental pluripotency associated factor 4
ENCODE: Encyclopedia of DNA Elements
epiFASD: Epigenetics of FASD
FASD: Fetal alcohol spectrum disorder
FDR: False discovery rate
FOXP2: Transcription factor forkhead box P2
FuGu: Biomedicum Functional Genomics Unit
GO: Gene Ontology
GW: Gestational week
GWAM: Genome-wide average DNA methylation
HC: Head circumference
hESC: Human embryonic stem cell
IL: Induction of labor
MAF: Minor allele frequency
mCpH: Methylated non-CpG site
mRNA-seq: 3’mRNA sequencing
N_shelf: North shelf
N_shore: North shore
PAE: Prenatal alcohol exposure
PCA: Principal component analysis
qPCR: Quantitative PCR
Q-Q: Quantile-Quantile
S_shelf: South shelf
S_shore: South shore
SD: Standard deviation
SVD: Singular value decomposition
TACR3: Tachykinin Receptor 3
TBC1D5: TBC1 domain family member 5
TSS1500: 1500 bp upstream of transcription start site
TSS200: 200 bp upstream of transcription start site
UCSC: University of California, Santa Cruz
UTR: Untranslated region
WBC: White blood cell
