# Supplementary material for: Chromatin modifier developmental pluripotency associated factor 4 (DPPA4) is a candidate gene for alcohol-induced developmental disorders
Source: BMC Med. 2022 Dec 30;20:495. doi: 10.1186/s12916-022-02699-1 (PMC9801659; doi:10.1186/s12916-022-02699-1)
Supplement: Supplementary file 2 — Additional file 2: Figure S1. Timing and amount of maternal alcohol consumption in categories Figure S2. Characterization of hESC differentiation into germ layer cells Figure S3. Q-Q plots of genome-wide DNAm (adjusted by smoking and sex) and sensitivity analyses (adjusted by sex, maternal age, mode of delivery, and parity) Figure S4. SVD plots of sensitivity analysis for candidate genes Figure S5. PCA for PAE-associated DMPs in placenta Figure S6. Effect sizes of candidate gene DMPs in genome-wide DNAm and sensitivity analyses Figure S7. Schematic figure about PAE-associated DMR at IGF2/H19 locus in placenta Figure S8. Cell-type composition in placental samples Figure S9. GWAM comparison between control and PAE placentas Figure S10. GWAM comparison between alcohol-exposed and control hESCs Figure S11. Pathway analysis of DMPs in hESCs Figure S12. Pathway analysis of DMRs in hESCs Figure S13. Pathway analysis of differentially expressed genes in hESCs. [file 12916_2022_2699_MOESM2_ESM.pdf]

Additional file 2:

Supplementary figures for
Chromatin modifier *developmental pluripotency associated factor 4* (*DPPA4*) is a candidate gene for alcohol-induced developmental disorders

Figure S1: Timing and amount of maternal alcohol consumption in categories

Figure S2: Characterization of hESC differentiation into germ layer cells

Figure S3: Q-Q plots of genome-wide DNAm (adjusted by smoking and sex) and sensitivity analyses (adjusted by sex, maternal age, mode of delivery, and parity)

Figure S4: SVD plots of sensitivity analysis for candidate genes

Figure S5: PCA for PAE-associated DMPs in placenta

Figure S6: Effect sizes of candidate gene DMPs in genome-wide DNAm and sensitivity analyses

Figure S7: Schematic figure about PAE-associated DMR at *IGF2/H19* locus in placenta

Figure S8: Cell-type composition in placental samples

Figure S9: GWAM comparison between control and PAE placentas

Figure S10: GWAM comparison between alcohol-exposed and control hESCs

Figure S11: Pathway analysis of DMPs in hESCs

Figure S12: Pathway analysis of DMRs in hESCs

Figure S13: Pathway analysis of differentially expressed genes in hESCs

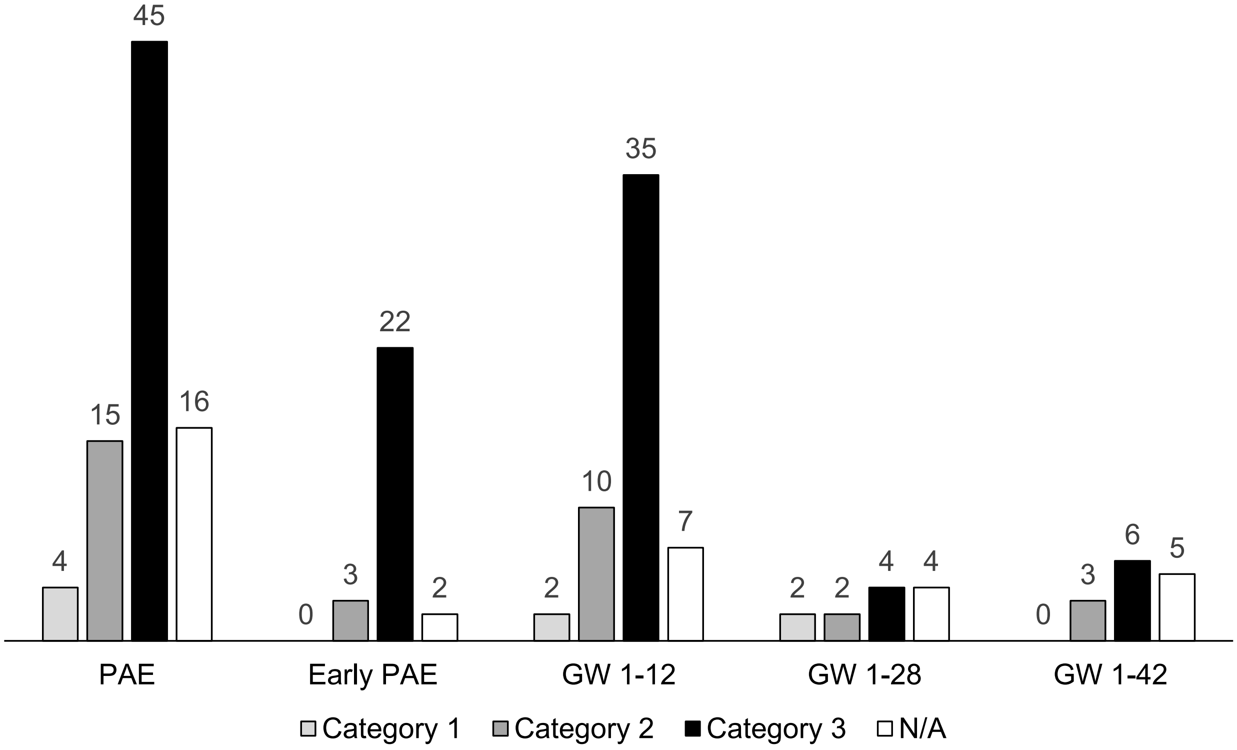

Supplementary figure 1: Timing and amount of maternal alcohol consumption in categories. Visual presentation of the timing and amount of alcohol consumption of mothers in the all PAE group (*n* = 80), early PAE subgroup (*n* = 27), and during gestational week (GW) 1–12, GW 1–28, and GW 1–42. Categories for maternal alcohol consumption: AUDIT scores 1–5 suggest low-risk consumption or < 7 alcohol units consumed per week (ad) cause low risk for morbidity and mortality for non-pregnant women (category 1), AUDIT scores 6–13 suggest hazardous or harmful alcohol consumption or 7–11 ad cause moderate risk for morbidity and mortality for non-pregnant women (category 2), and AUDIT scores 14–40 indicate the likelihood of alcohol dependence (moderate-severe alcohol use disorder) or ≥ 12 ad cause high risk for morbidity and mortality for non-pregnant women (category 3). The categories for maternal alcohol consumption were defined by combining information from previous publications[18,19,20].

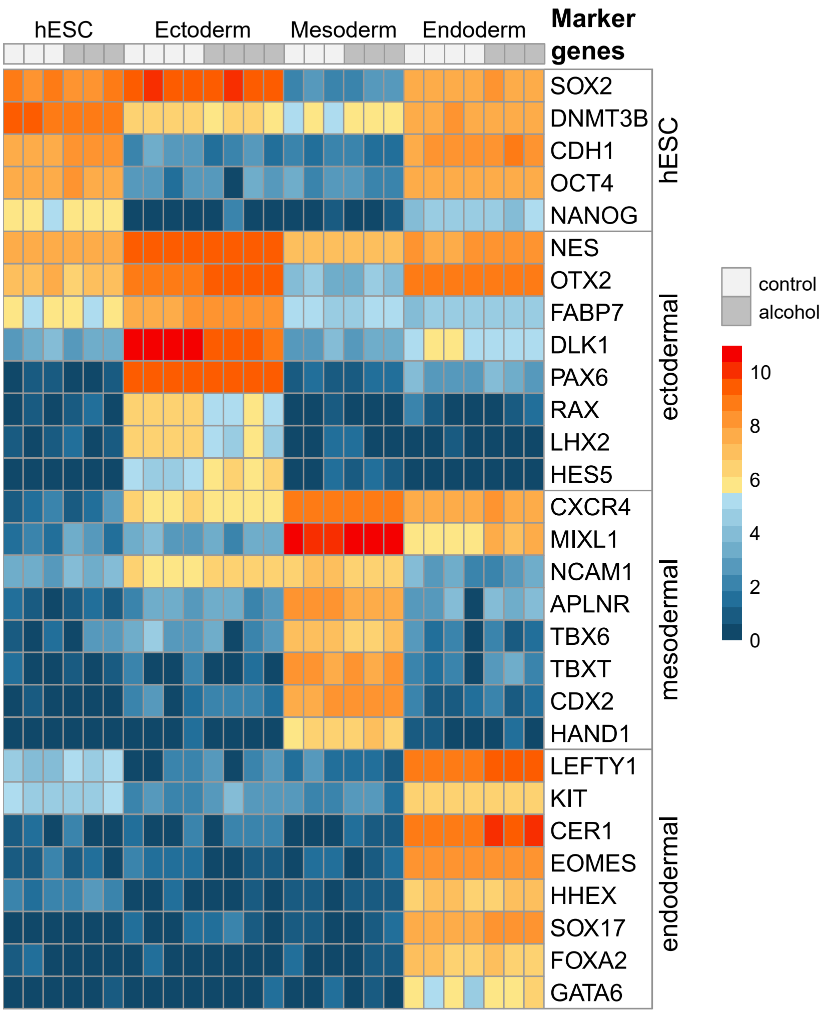

Supplementary figure 2: Characterization of hESC differentiation into germ layer cells. Heatmap visualization of cell type-specific gene expression profiles in undifferentiated control and alcohol-exposed hESCs (*n* = 3/condition) as well as in differentiated control and alcohol-exposed ectodermal (*n* = 4/condition), mesodermal (*n* = 3/condition), and endodermal (control *n* = 4 and alcohol-exposed *n* = 3) cells.

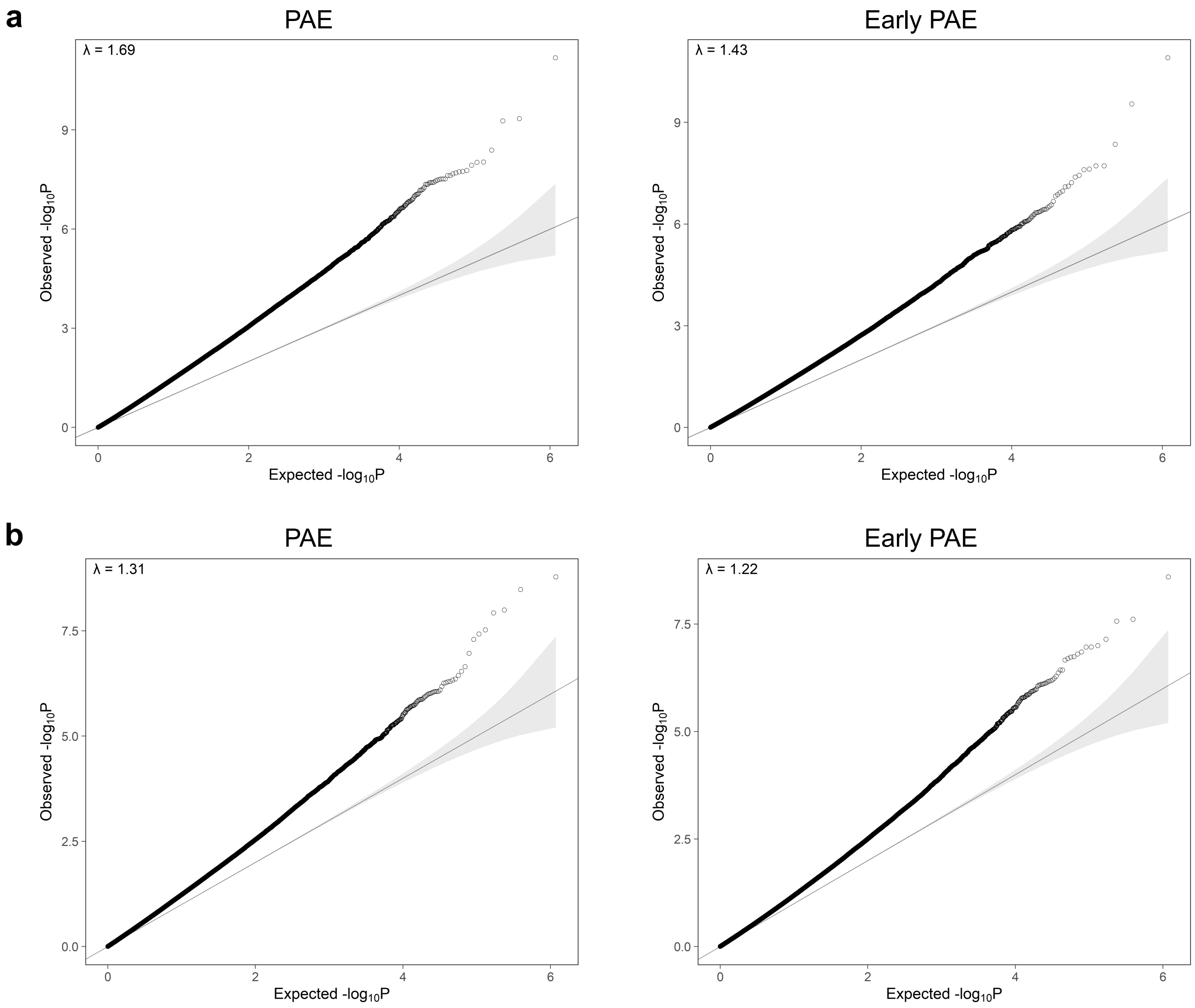

Supplementary figure 3: Q-Q plots of genome-wide DNAm (adjusted by smoking and sex) and sensitivity analyses (adjusted by sex, maternal age, mode of delivery, and parity). a Q-Q plots of genome-wide DNAm analysis of control (n = 66) and PAE (n = 69) placentas as well as control (n = 66) and early PAE (n = 27) placentas. **b** Q-Q plots of sensitivity analysis of control (n = 66) and selected PAE (n = 8) placentas as well as control (n = 66) and selected early PAE (n = 4) placentas.

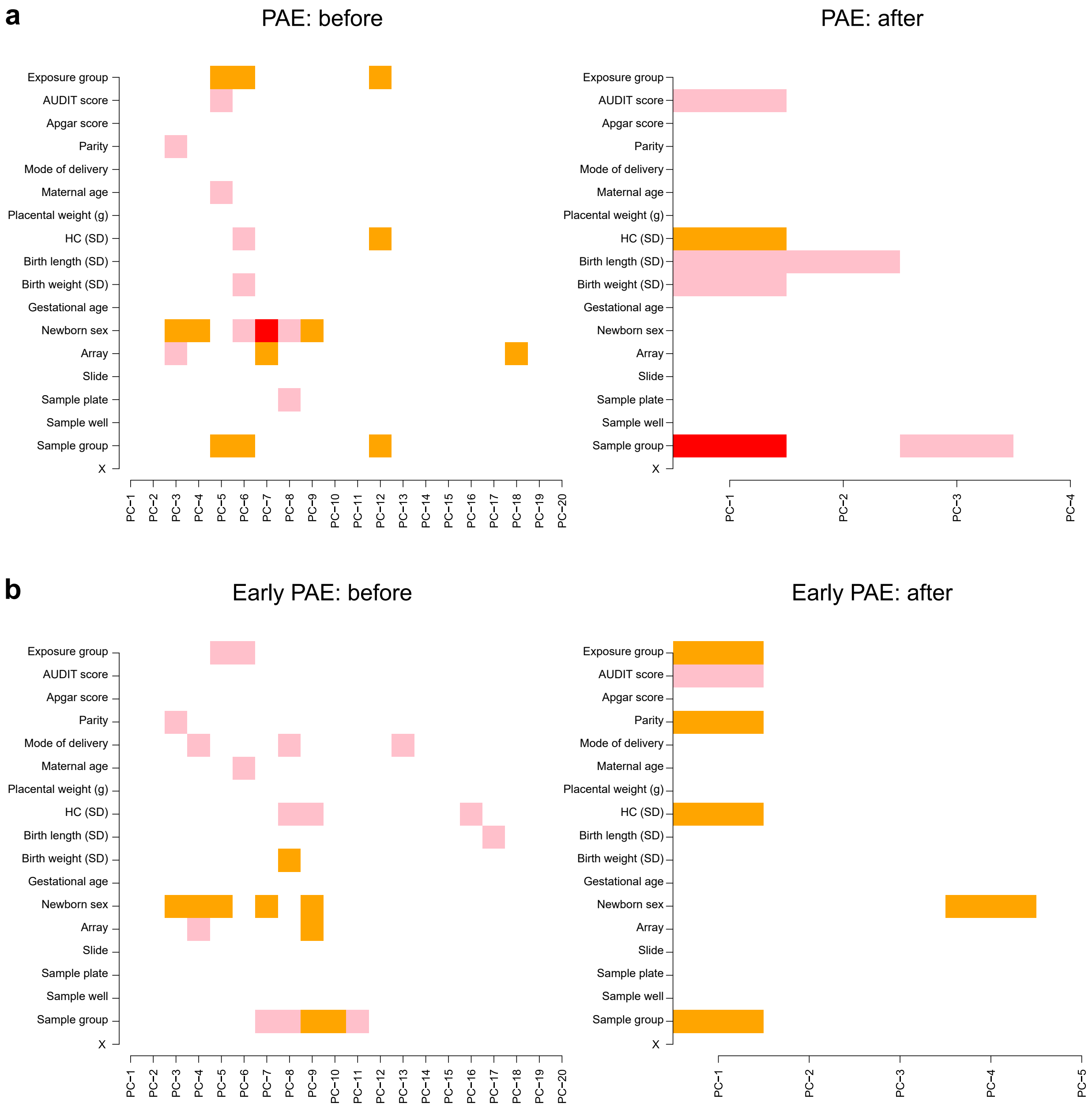

Supplementary figure 4: SVD plots of sensitivity analysis for candidate genes. a SVD plots before and after the sensitivity analysis for candidate genes of selected placental control (n = 66) and PAE (n = 8) samples. **b** SVD plots before and after sensitivity analysis for candidate genes of selected placental control (n = 66) and early PAE (n = 4) samples. Exposure group: control (n = 66) and early PAE (n = 4) placental samples. Sample group: control (n = 66) and PAE (n = 8) placental samples. Mode of delivery: vaginal delivery, elective caesarean section, or emergency caesarean section.

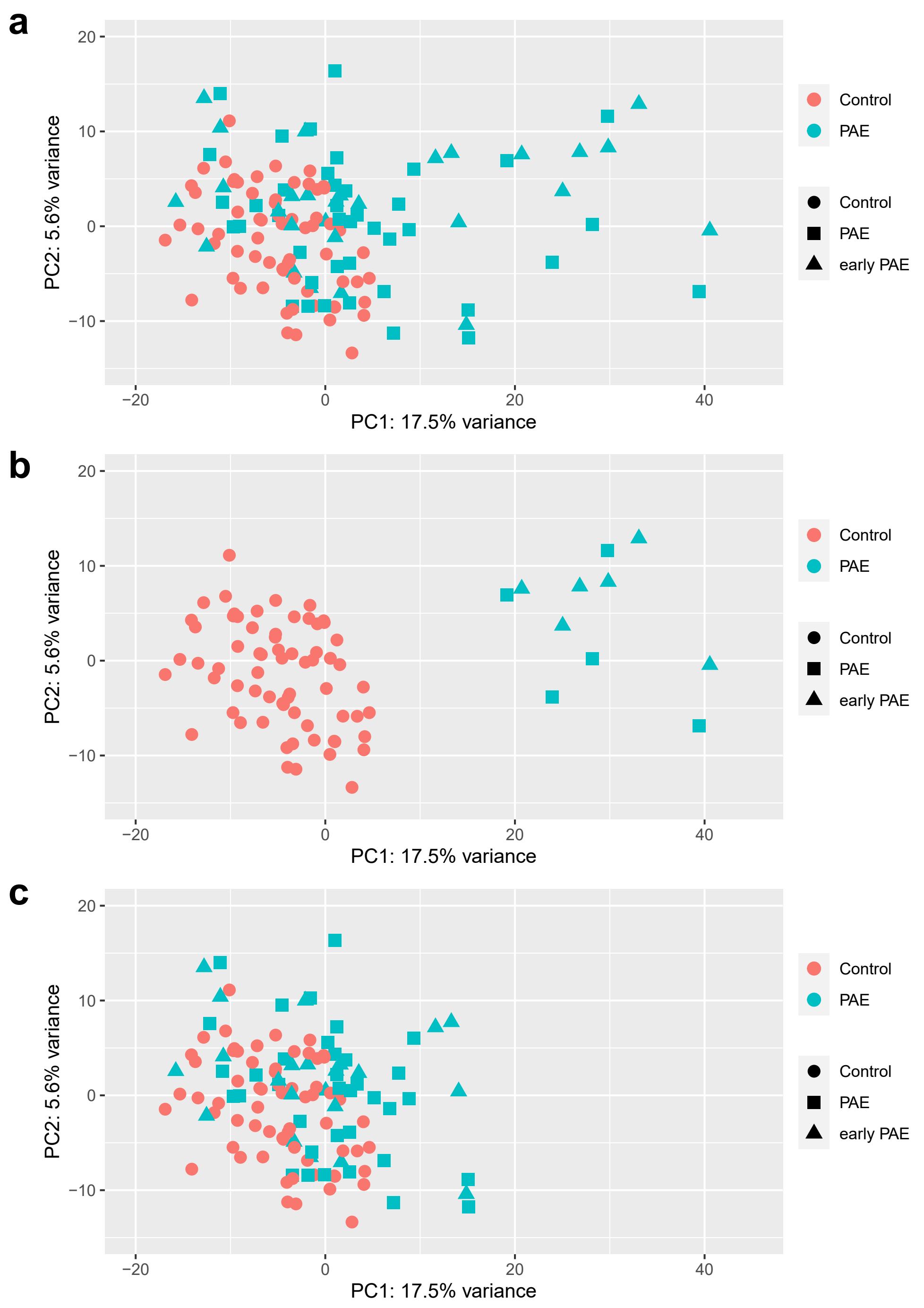

Supplementary figure 5: PCA for PAE-associated DMPs in placenta. Scatter plot of the first two principal components (PC1 and PC2) has been displayed in separate figures of **a** control (*n* = 66) and PAE (*n* = 69) placentas, **b** control placentas (*n* = 66) as well as PAE placentas that were not exposed to smoking during pregnancy (*n* = 11), and **c** control placentas (*n* = 66) as well as PAE placentas that were exposed to smoking during pregnancy (*n* = 58). Red dots represent control, blue squares PAE, and blue triangles early PAE placental samples.

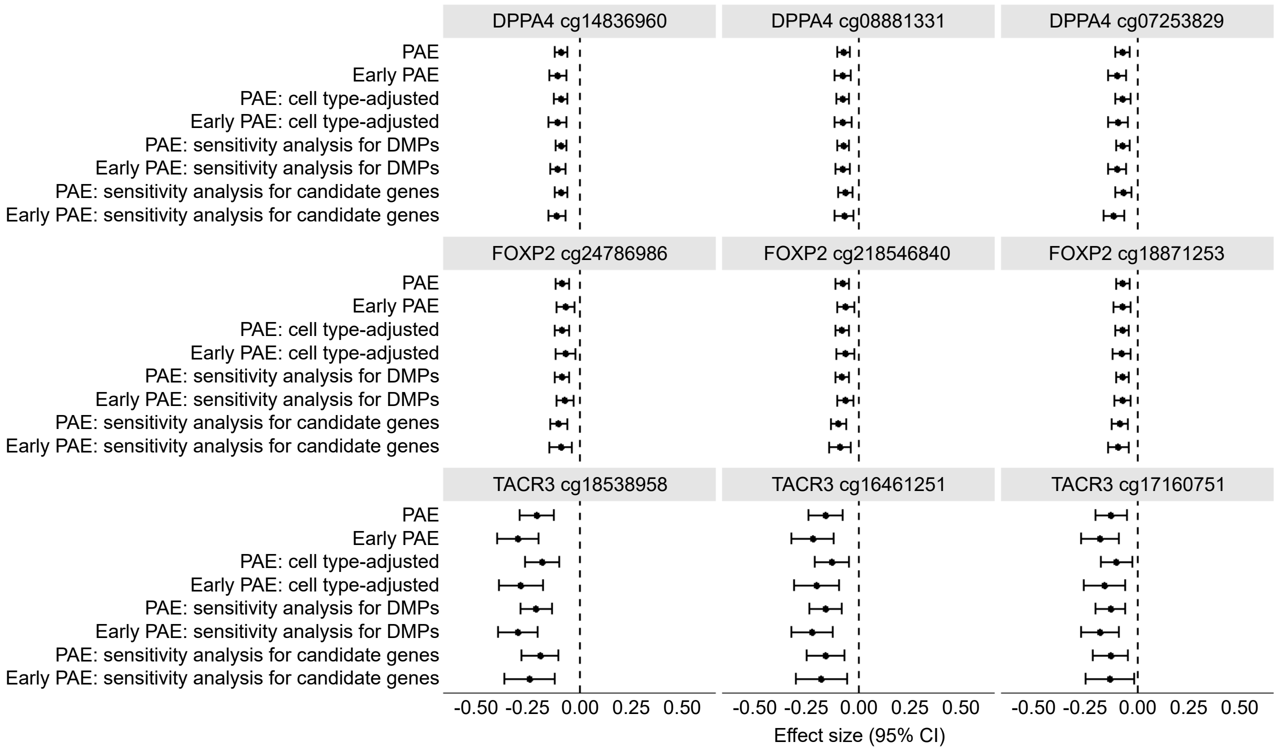

Supplementary figure 6: Effect sizes of candidate gene DMPs in genome-wide DNAm and sensitivity analyses. Forest plot visualization of the effect sizes (mean $\Delta\beta$) of candidate gene DMPs in genome-wide DNAm analyses of PAE and early PAE placentas with and without a cell type adjustment (control n = 66, PAE n = 69, early PAE n = 27) as well as in sensitivity analyses for DMPs (control n = 66, PAE n = 11, early PAE n = 6) and candidate genes (control n = 66, PAE n = 8, early PAE n = 4). Three DMPs of each candidate gene with the largest effect sizes are presented. 95% Confidence Interval: 95% CI.

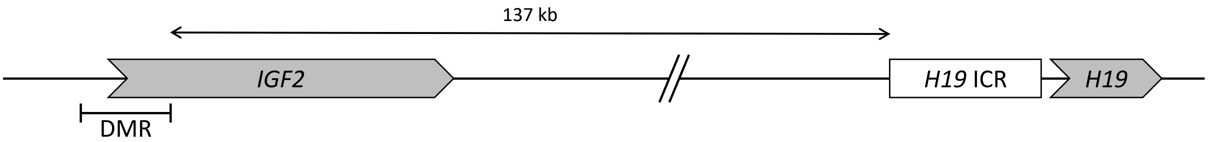

Supplementary figure 7: Schematic figure about PAE-associated DMR at *IGF2/H19* locus in placenta. DMR (chr11:2,161,544-2,163,299) locates at the first exon of *IGF2* transcript variant 3 (NM_001127598.3) and 5 (NM_001291862.3), and is located 137 kb from *H19* imprinting control region (ICR) (chr11:2,019,975-2,024,739) at chromosome 11 (GRCh37/hg19).

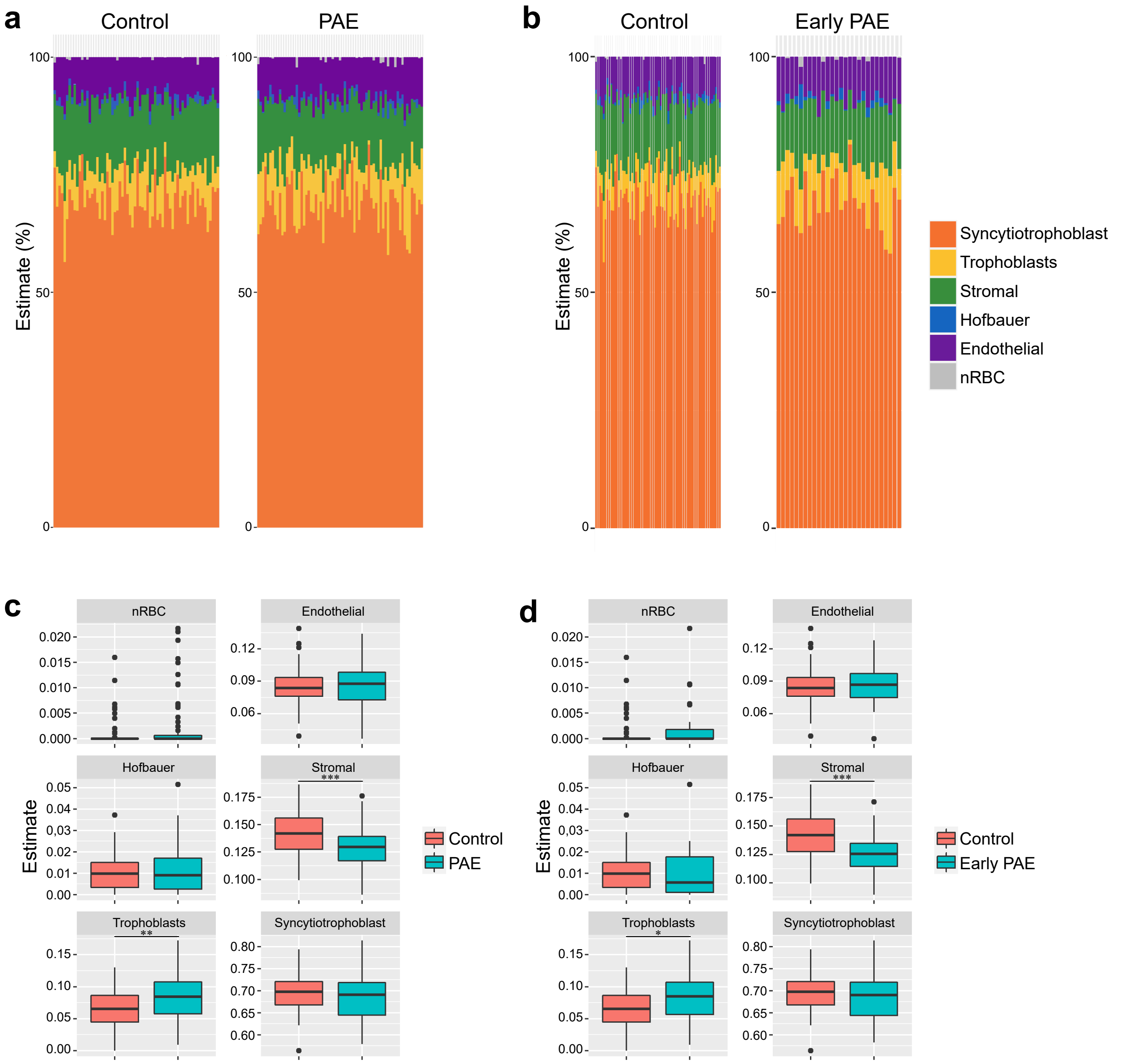

Supplementary figure 8: Cell-type composition in placental samples. a Cell-type composition in control and PAE samples. **b** Cell-type composition in control and early PAE samples. **c** Box plot presentation of cell-type composition in control and PAE samples. **d** Box plot presentation of cell-type composition in control and early PAE samples. *$P < 0.01$ and **$P < 0.01$, Wilcoxon test, and ***$P < 0.001$, two-tailed Student's *t*-test. Control $n = 66$, PAE $n = 69$, and early PAE $n = 18$.

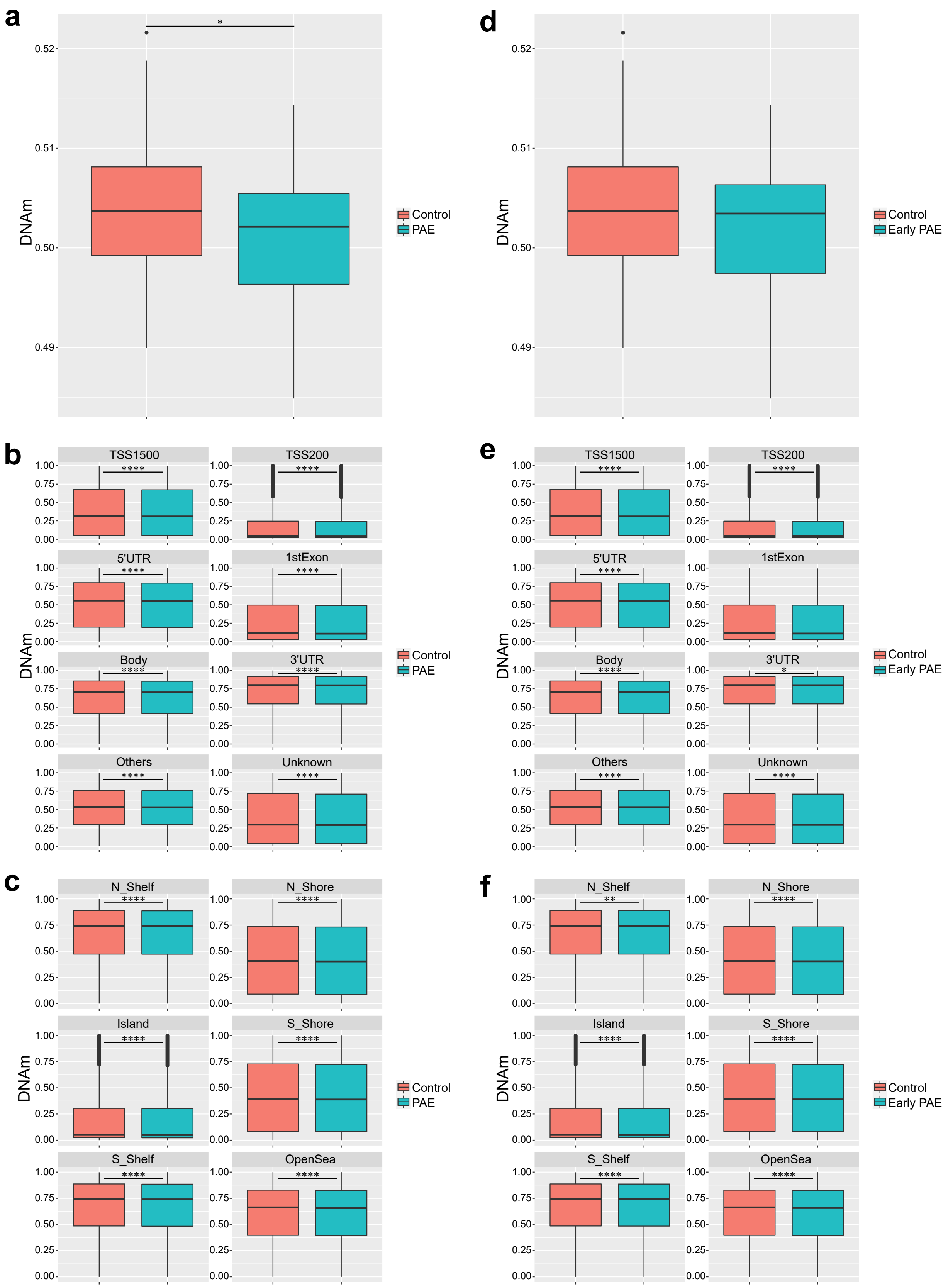

Supplementary figure 9: GWAM comparison between control and PAE placentas. a Comparison of DNAm in all probes between control and PAE placentas. **b** Comparison of probe

DNAm in relation to gene between control and PAE placentas. **c** Comparison of probe DNAm in relation to CpG island between control and PAE placentas. **d** Comparison of DNAm in all probes between control and early PAE placentas. **e** Comparison of probe DNAm in relation to gene between control and early PAE placentas. **f** Comparison of probe DNAm in relation to CpG island between control and early PAE placentas. DMPs were divided to hypo- and hypermethylated subgroups, which were further grouped according to the genomic location based on UCSC database. If the location information was missing, DMP was marked as 'unknown'. In the case of multiple location entries, group 'others' was used. *$P < 0.01$ and **$P < 0.001$, two-tailed Student's *t*-test, and ****$P < 0.0001$, two-tailed Student's *t*-test or Wilcoxon test. Control $n = 66$, PAE $n = 69$, and early PAE $n = 27$. Abbreviations TSS1500: 1500 bp upstream of transcription start site, TSS200: 200 bp upstream of TSS, UTR: untranslated region, N_shelf: north shelf, N_shore: north shore, S_shore: south shore, S_shelf: south shelf.

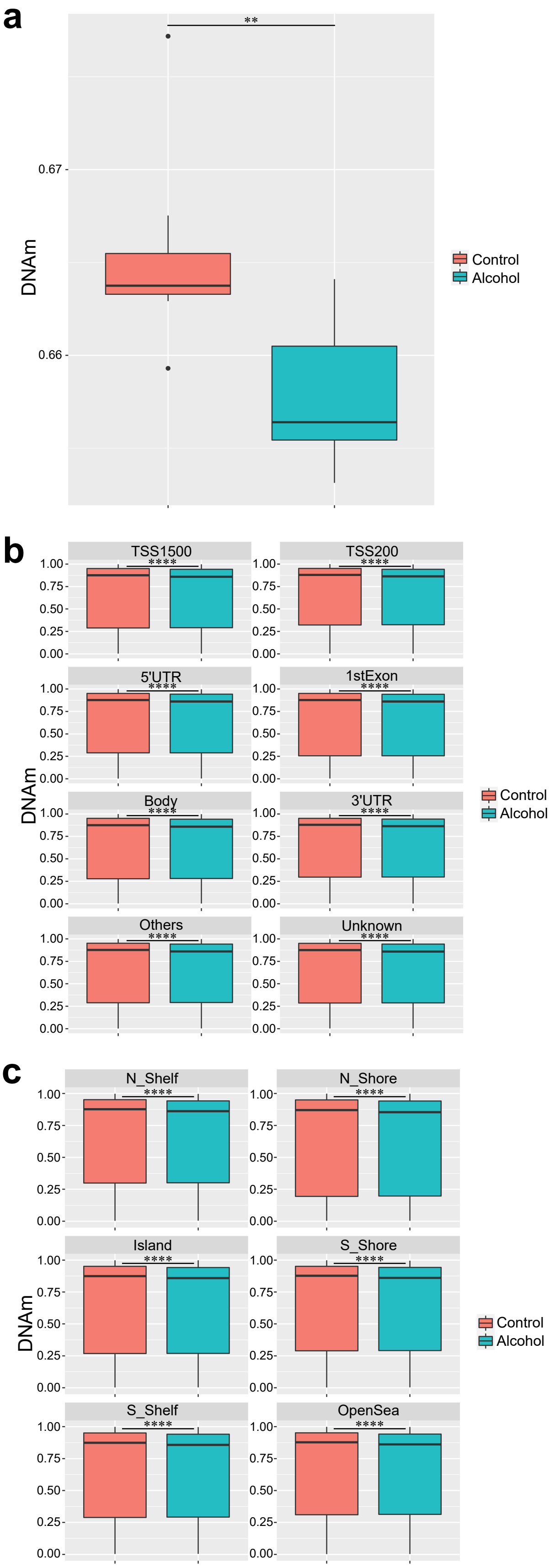
a
**
0.67
0.66
DNAm
Control
Alcohol
b
TSS1500
TSS200
5'UTR
1stExon
Body
3'UTR
Others
Unknown
****
1.00
0.75
0.50
0.25
0.00
DNAm
Control
Alcohol
c
N_Shelf
N_Shore
Island
S_Shore
S_Shelf
OpenSea
****
1.00
0.75
0.50
0.25
0.00
DNAm
Control
Alcohol

Supplementary figure 10: GWAM comparison between alcohol-exposed and control hESCs. **a** Comparison of DNAm in all probes between control and alcohol-exposed hESCs. **b** Comparison of probe DNAm in relation to gene between control and alcohol-exposed hESCs. **c** Comparison of probe DNAm in relation to CpG island between control and alcohol-exposed hESCs. DMPs were divided to hypo- and hypermethylated subgroups, which were further grouped according to the genomic location based on UCSC database. If the location information was missing, DMP was marked as 'unknown'. In the case of multiple location entries, group 'others' was used. $^{**}P < 0.01$, two-tailed Student's *t*-test and $^{****}P < 0.0001$, Wilcoxon test. Control and alcohol-exposed hESCs $n = 8$, respectively. Abbreviations TSS1500: 1500 bp upstream of transcription start site, TSS200: 200 bp upstream of TSS, UTR: untranslated region, N_shelf: north shelf, N_shore: north shore, S_shore: south shore, S_shelf: south shelf.

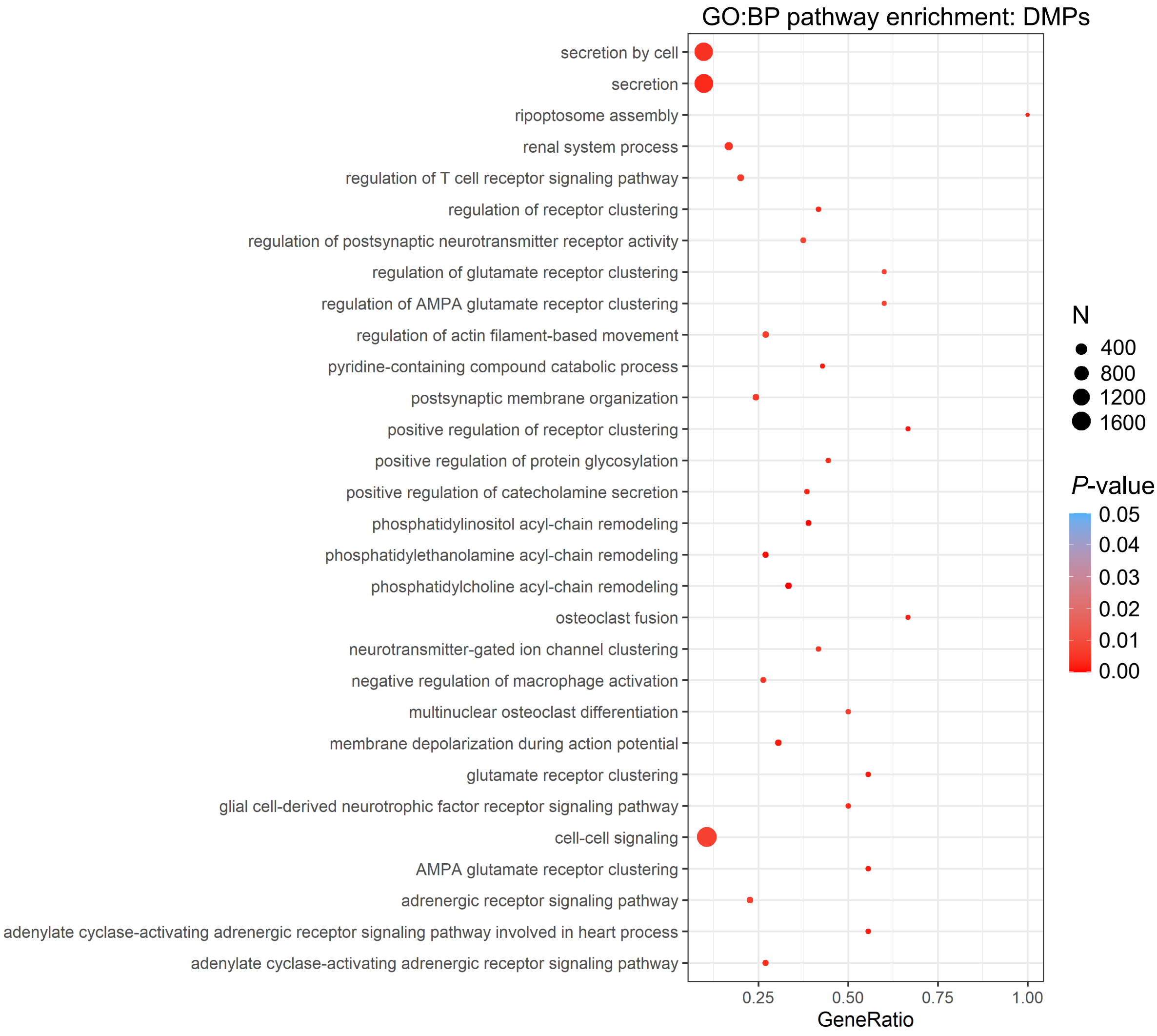

Supplementary figure 11: Pathway analysis of DMPs in hESCs. Enriched terms identified in GO:BP enrichment analysis of alcohol-induced DMPs in hESCs ($P < 0.05$). The 30 most significant pathways are shown.

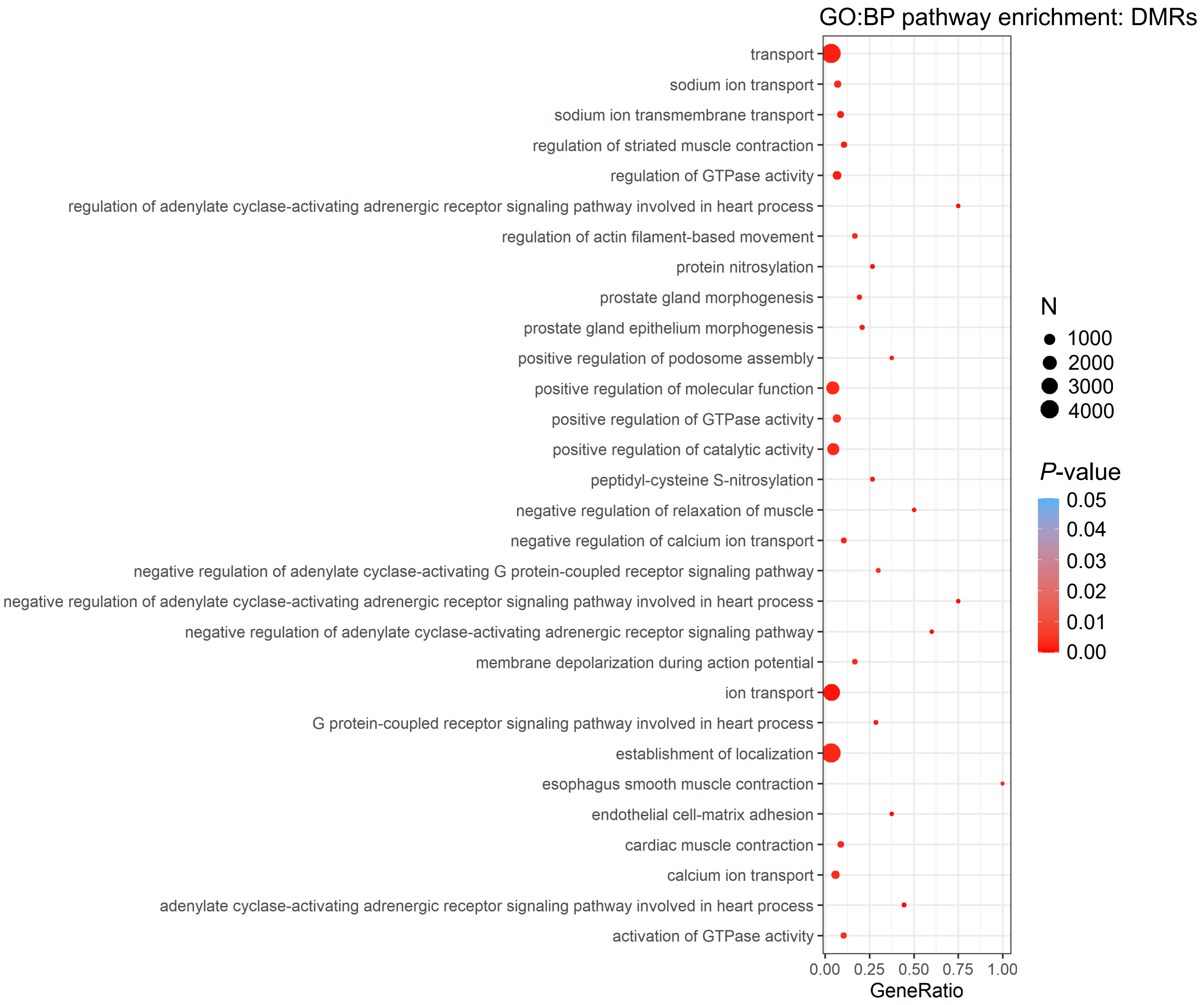

Supplementary figure 12: Pathway analysis of DMRs in hESCs. Enriched terms identified in GO:BP enrichment analysis of alcohol-induced DMRs in hESCs (*P* < 0.05). The 30 most significant pathways are shown.

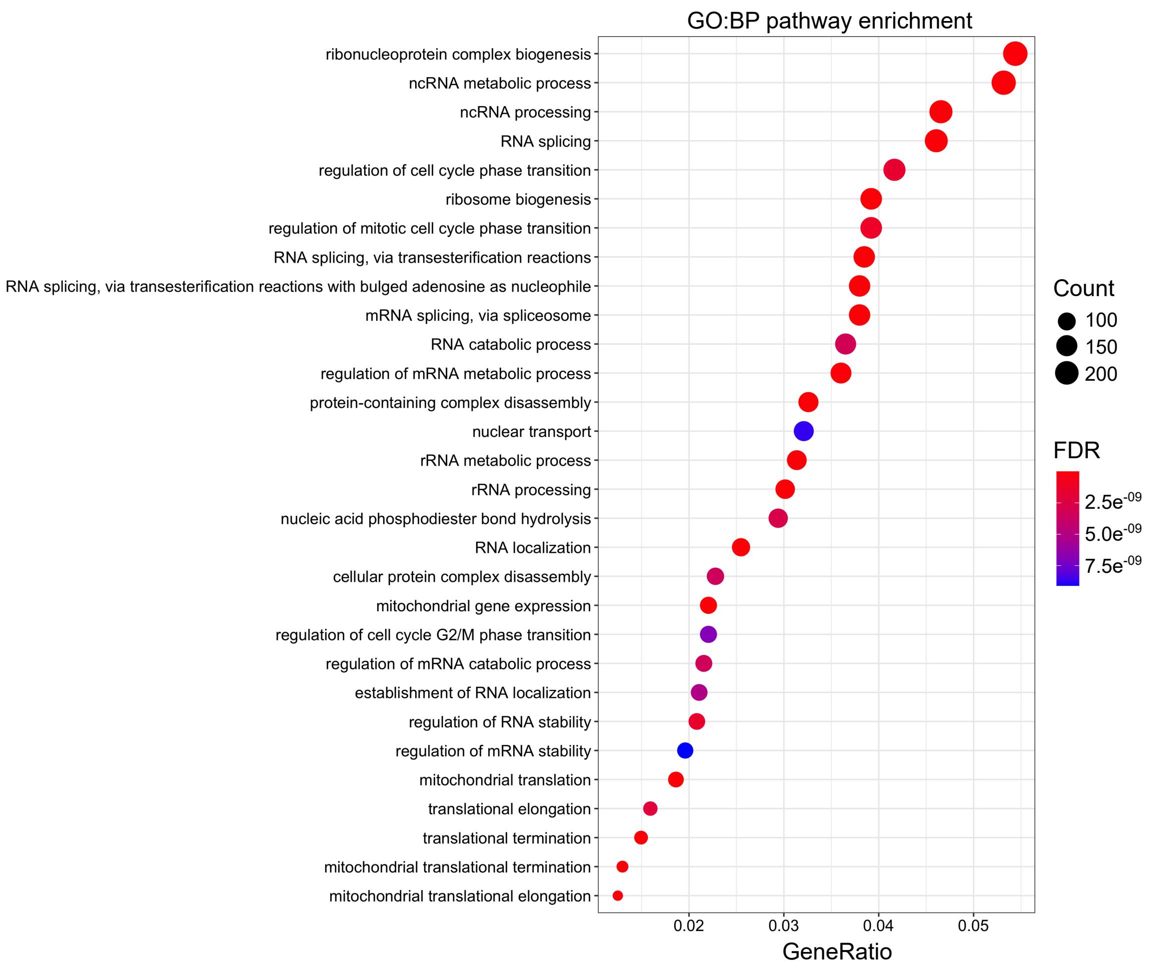

Supplementary figure 13: Pathway analysis of differentially expressed genes in hESCs. Significantly enriched terms identified in GO:BP enrichment analysis of alcohol-induced differentially expressed genes in hESCs (FDR-corrected *q*-value < 0.05). The 30 most significant pathways are shown.
